# Hybrid cardiovascular imaging. A clinical consensus statement of the european association of nuclear medicine (EANM) and the european association of cardiovascular imaging (EACVI) of the ESC

**DOI:** 10.1007/s00259-024-06946-w

**Published:** 2024-10-22

**Authors:** Federico Caobelli, Marc R. Dweck, Domenico Albano, Olivier Gheysens, Panagiotis Georgoulias, Stephan Nekolla, Olivier Lairez, Lucia Leccisotti, Marc Lubberink, Samia Massalha, Carmela Nappi, Christoph Rischpler, Antti Saraste, Fabien Hyafil

**Affiliations:** 1 Department of Nuclear Medicine, University Hospital Bern, University of Bern, Freiburgstrasse 18, 3010 Bern, Switzerland; 2 Centre for Cardiovascular Sciences, University of Edinburgh, Edinburgh, UK; 3 Department of Nuclear Medicine, University of Brescia, Brescia, Italy; 4 Department of Nuclear Medicine, Cliniques Universitaires Saint-Luc and Institute of Clinical and Experimental Research (IREC), Université Catholique de Louvain, Brussels, Belgium; 5 Department of Nuclear Medicine, Faculty of Medicine, University of Thessaly, University Hospital of Larissa, Larissa, Greece; 6 Department of Nuclear Medicine, School of Medicine, Klinikum Rechts Der Isar, Technical University of Munich, Munich, Germany; 7 National Institute of Health and Medical Research (INSERM), I2MC, U1297, Toulouse, France; 8 Department of Nuclear Medicine, Fondazione Policlinico Universitario A. Gemelli IRCCS and Università Cattolica del Sacro Cuore, Rome, Italy; 9 Department of Surgical Sciences/Nuclear Medicine & PET, Uppsala University, Uppsala, Sweden; 10 Rambam Health Campus, Haifa, Israel; 11 Department of Advanced Biomedical Sciences, University Federico II, Via Pansini 5, 80131 Naples, Italy; 12 Department of Nuclear Medicine, Klinikum Stuttgart, Stuttgart, Germany; 13 Turku PET Centre, Turku University Hospital and University of Turku, Turku, Finland; 14 Department of Nuclear Medicine, AP-HP, European Hospital Georges-Pompidou, University of Paris-Cité, 75015 Paris, France

**Keywords:** Hybrid imaging, Cardiovascular imaging, PET/MR, PET/CT

## Abstract

Hybrid imaging consists of a combination of two or more imaging modalities, which equally contribute to image information. To date, hybrid cardiovascular imaging can be performed by either merging images acquired on different scanners, or with truly hybrid PET/CT and PET/MR scanners. The European Association of Nuclear Medicine (EANM), and the European Association of Cardiovascular Imaging (EACVI) of the European Society of Cardiology (ESC) aim to review clinical situations that may benefit from the use of hybrid cardiac imaging and provide advice on acquisition protocols providing the most relevant information to reach diagnosis in various clinical situations.

## Preamble

The European Association of Cardiovascular Imaging (EACVI) of the European Society of Cardiology (ESC) represents the world-leading network of cardiovascular imaging experts, providing a unified community gathering four imaging modalities under one entity (echocardiography, cardiovascular magnetic resonance, nuclear cardiology and cardiac computed tomography).

The European Association of Nuclear Medicine (EANM) is a professional non-profit medical association that facilitates communication worldwide between individuals pursuing clinical and research excellence in nuclear medicine. The EANM was founded in 1985. EACVI and EANM members are physicians, technologists, and scientists specializing in the research and practice of cardiovascular imaging.

Each consensus paper, representing a clinical consensus statement by the EACVI/EANM, has undergone a thorough consensus process in which it has been subjected to extensive review. The EACVI and EANM recognize that the safe and effective use of diagnostic nuclear medicine imaging requires specific training, skills, and techniques, as described in each document. Reproduction or modification of the published consensus paper by those entities not providing these services is not authorized.

The clinical consensus statement is an educational tool designed to assist practitioners in providing appropriate care for patients. They are not inflexible rules or requirements of practice and are not intended, nor should they be used, to establish a legal standard of care. For these reasons and those set forth below, both the EACVI and the EANM caution against the use of these consensus papers in litigation in which the clinical decisions of a practitioner are called into question.

The ultimate judgment regarding the propriety of any specific procedure or course of action must be made by the physician or medical physicist in light of all the circumstances presented. Thus, there is no implication that an approach differing from the consensus papers, standing alone, is below the standard of care. To the contrary, a conscientious practitioner may responsibly adopt a course of action different from that set forth in the consensus paper when, in the reasonable judgment of the practitioner, such course of action is indicated by the condition of the patient, limitations of available resources, or advances in knowledge or technology subsequent to publication of the consensus paper.

The practice of medicine includes both the art and the science of the prevention, diagnosis, alleviation, and treatment of disease. The variety and complexity of human conditions make it impossible to always reach the most appropriate diagnosis or to predict with certainty a particular response to treatment.

Therefore, it should be recognized that adherence to these consensus papers will not ensure an accurate diagnosis or a successful outcome. All that should be expected is that the practitioner will follow a reasonable course of action based on current knowledge, available resources, and the needs of the patient to deliver effective and safe medical care. The sole purpose of these consensus papers is to assist practitioners in achieving this objective.

## Introduction

Hybrid imaging consists of a combination of two or more imaging modalities, which equally contribute to image information [1]. In recent years, the use of hybrid cardiovascular imaging has increased in parallel with the development of integrated systems that combine morphological and anatomic imaging from computed tomography (CT) or magnetic resonance (MR) with molecular/functional imaging provided by nuclear medicine modalities such as positron emission tomography (PET) or single-photon emission computed tomography(SPECT) [2–5].

To date, hybrid cardiovascular imaging can be performed by either merging images acquired on different scanners, or with truly hybrid PET/CT and PET/MR scanners. Of note, the image quality provided by CT or MRI scanners integrated within hybrid imaging systems has now become close to that offered by stand-alone scanners [6].

Cardiac hybrid imaging has the advantage of providing as a one-stop shop non-invasive imaging procedures with reduced acquisition time. Furthermore, the combination of both morphological and functional/molecular information can improve overall diagnostic accuracy and risk stratification e.g. in patients with suspected coronary artery disease (CAD) [7–11] and cardiac inflammatory or infectious diseases [5]. Hybrid imaging is, however associated with increased radiation exposure and should only be used if there is a clear medical benefit for patients and radiation dose minimization. In this document, experts from the European Association of Nuclear Medicine (EANM), and the European Association of Cardiovascular Imaging (EACVI) of the ESC have reviewed clinical indications that may benefit from the use of hybrid cardiac imaging and provided optimal acquisition protocols for each situation.

## Hybrid imaging with CT

### CT protocols

In the context of hybrid imaging, there are three main categories of CT protocols, all typically being acquired during a single breath-hold and with possible integration of the electrocardiogram (ECG) signal in order to minimize cardiac-motion artifacts during the scan. Coronary Artery Calcium Scoring (CACS) CT is a non-contrast, ECG-gated CT scan that is used to measure the amount of calcium in the coronary arteries. By contrast, CT coronary angiography (CCTA) is acquired during the arterial phase of a bolus injection of iodinated contrast media (around 100 ml). Acquisition of the coronary vessels in held-inspiration allows assessment of luminal stenoses and coronary plaque with minimum artifacts attributed to coronary artery motion. Furthermore, CT angiography is also commonly used (with a very similar protocol, i.e. contrast-enhanced ECG-gated non-coronary CT angiography) to image the aorta, cardiac implants and pulmonary veins and to evaluate valvular anatomy prior to Transcatheter Aortic Valve Implantation (TAVI) [12] or in the assessment of infective endocarditis. In addition, low-dose CT without breathing instruction and without ECG-gating are commonly performed to correct for attenuation of tomographic emission acquisitions either from SPECT or PET (Table 1).

**Table 1:** Technical parameters of different CT protocols

	CT for attenuation correction	Nonenhanced CT for anatomical localization	ECG-gated nonenhanced CT (CACS)	CT angiography	ECG-gated CT angiography
mAs	20 to 40	20	20 to 40	150 to 350	150 to 350
kVp	80 to 100	80	100 to 120	80 to 120	80 to 120
Slice thickness	5 mm	5 mm	2 mm	1–2 mm	0.75 mm
Matrix size	512 × 512	128 × 128	512 × 512	512 × 512	512 × 512
Contrast medium	no	no	no	Yes, 4–5 ml/min	Yes, 4–5 ml/min

CACS can be obtained with a 16-slice CT scanner, but a 64-slice scanner or more advanced is required to performe CCTA. It should be noted that the CT components included in hybrid devices may be technically less advanced than stand–alone systems due to the system’s size in addition to financial considerations.

**Fig. 1:**
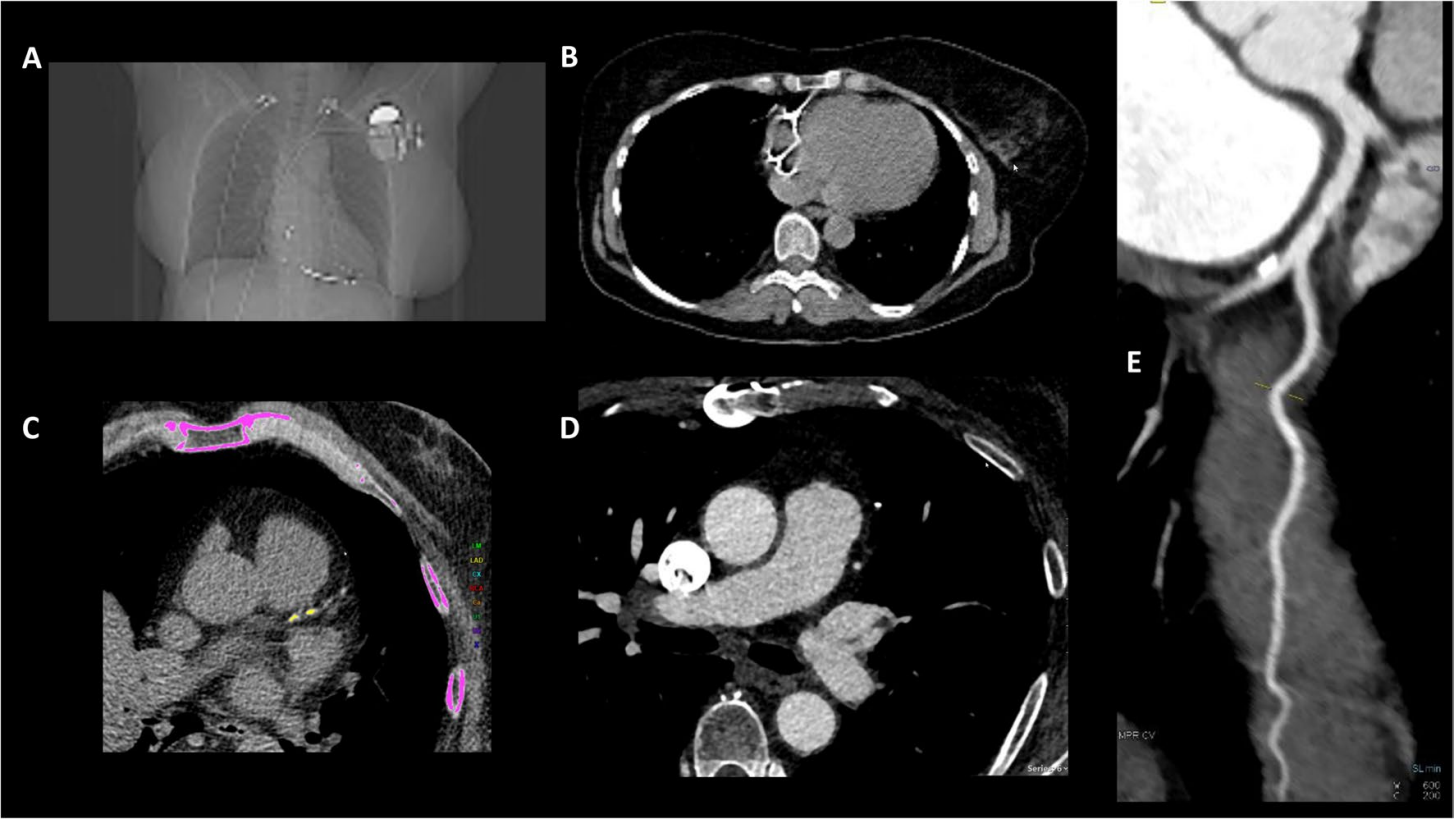
Different CT protocols commonly used in cardiovascular imaging. A: non-enhanced CT for the anatomical localization; B: non-enhanced CT for attenuation correction (AC); C: non-enhanced, ECG-gated CT for the assessment of coronary artery calcium score (CACS); D: CT-based angiography with contrast medium (CTA); E: CT-based, ECG-gated coronary angiography (CCTA)

Contraindications for CCTA include known severe allergic reactions or anaphylaxis to iodinated contrast material, inability to co-operate with scan acquisition/breath-hold instructions, and an unstable clinical condition [13]. Iodinated contrast material is associated with a risk of worsening of renal insufficiency that needs to be considered in patients with impaired renal function according to local protocols [13]. Pregnancy is often a contraindication to modalities involving radiation exposure, due to potential effects of radiation to fetus [13, 14]. In addition to the above contraindications, there are patient-related variables that affect the diagnostic accuracy of CCTA and CACS especially if performed on a hybrid system, wherein the CT component may be less efficient than stand-alone, fast CT scanners. Specifically, an irregular heart rate (atrial fibrillation or frequent ectopic beats), a fast heart beat that cannot be rate-controlled, severe obesity and the inability to perform breath-holding are associated with increased likelihood of non-diagnostic image quality due to motion artifacts [13]. An overview of the different CT protocols is displayed in Fig. 1. Since the CT component in hybrid systems can be  less advanced than stand-alone, new generation CT scanners, advice may partly differ to those published for stand-alone CT modalities [15, 16].

#### Radiation exposure

In PET/CT and SPECT/CT systems, both nuclear imaging and CT components contribute to ionizing radiation exposure. Special attention should be taken to minimize radiation burden. As mentioned, the image quality and the radiation dose are a function of the used CT and PET/SPECT system, but patient-specific factors such as chest diameter and scan range may contribute as well. Although the range of exposures is not as large as in early studies investigating radiation doses from different CT scanner generations [17], the individual radiation exposure can vary substantially with the CT system, its age being a relevant indicator [18]. For example, using commonly available single-source 64-slice CT scanners with a prospectively ECG-triggered step-and-shoot acquisition protocol, it is possible to consistently perform a CCTA with an absorbed radiation dose of 2 to 10 mSv [19]. CT scan for CACS evaluation adds less radiation (0.5–2 mSv) to the patient than CCTA [20]. Dedicated stand-alone cardiac CT systems for coronary and structural diseases might have more optimal radiation profile and imaging protocols for young individuals if available at the local hospital setting. Radiation exposure of patients depends not only from the type of scanner used, but also from other factors including patient geometry and cardiac rhythm. A detailed review on doses from ionizing radiation in cardiovascular CT summarizing data from large international cohorts was recently published by Kędzierski et al. [21]. In summary, image quality and patient radiation dose are influenced by a variety of factors some of which are hardware dependent whilst others can and should be optimized: the latter include decreasing the X-ray voltage, regulating the tube current with ECG, employing iterative and potentially deep learning reconstruction methods, limiting the scan range (avoiding the “safety margin”), utilizing prospective gating, implementing automatic exposure control, and finally managing heart rate [17, 21, 22].

#### Artifacts

There are several challenges associated with cardiac hybrid imaging. The merging of two distinct data sets, such as PET or SPECT and CT, into a single cohesive data set, which is referred to as image registration, can be challenging. The task requires accurate alignment of the two images in three-dimensions prior to clinical reading, which can be complicated by discrepancies in patient placement and movement, as well as disparities in the timing and duration of the imaging protocols. In the setting of hybrid imaging, this is further complicated by the fact that the PET or SPECT scatter and attenuation relies on accurate alignment between the two modalities [23–25]. Thus, strict quality control prior to clinical reading and, if necessary, motion correction and re-reconstruction are mandatory [26, 27]. In the latest years, several attempts have been done to minimize the possibility of incorrect data due to misregistration, especially if such misregistration was caused by patient’s movement [28, 29].

The introduction of digital “SPECT-only” systems using CZT detectors and employing a “dentist-chair” approach can reduce the presence of attenuation artifacts to some degree, and adding CT data, also acquired on other devices, increases readers’ confidence and diagnostic accuracy [30]. Differences in patient's position between the two scans can result in new “positional” artefacts, which need to be considered. There is evidence that the incidence of positional artifacts in females, individuals with higher BMI, and adenosine stress subsets is higher when the examination is performed in a semi-reclining position [31]**.**

An additional factor in hybrid cardiac imaging is the propagation of modality-specific artifacts into the other modality, in particular metal artifact associated with CT. With the increasing presence of implantable devices, this is becoming a more important problem [32–34]. While recent studies confirm the reliability of metal artefact reduction (MAR) algorithms on CT images to increase the image interpretability [35], it is worth noting that improvements in CT acquisition always take some time before they are migrated to hybrid systems.

### Myocardial perfusion imaging

To date, there are several indications for the use of CT in combination with myocardial perfusion imaging (MPI). The most widespread indication relies on the implementation of a low-dose CT for the correction of tissue attenuation (AC). While AC CT is currently mandatory for PET MPI, its use in SPECT MPI is to date not fully embedded in clinical protocols at all institutions. However, it was demonstrated in several papers that AC improves diagnostic accuracy in SPECT MPI, especially in obese patients [30, 36, 37].

The second indication is the assessment of CACS. The latter allows for the detection of coronary calcium burden with the potential to predict the presence of obstructive CAD in patients without known coronary stenosis, and to provide outcome data regarding future coronary events [38]. Beyond the key role of CACS in the primary CAD prevention, information derived from its evaluation are complementary to MPI [39]. Very high CACS may prompt invasive coronary evaluation in cases of unclear MPI, and high CACS associated with direct or indirect signs of left ventricular (LV) dysfunction after stress on ECG-gated images may increase suspicion of balanced myocardial ischemia, as seen in three-vessel CAD [40]. While a calculated CACS of 0 is consistent with a < 1% risk of having a significant coronary stenosis in the general population [41] and there is a significant association between increasing CACS and the prevalence of ischemia on MPI SPECT [42], a CACS score of 0 does not necessarily exclude obstructive disease in symptomatic patients. However, CACS helps to indicate the presence of non-obstructive coronary plaques without hemodynamic significance on MPI and may also identify a group of patients at increased risk during long-term follow-up, who may benefit from preventive treatment. An observational study indicates that in patients with moderate or high CACS (> 100), the use of statin therapy is associated with lower rate of cardiovascular events, whereas a CACS of 0 is linked to lower event risk and, possibly, little or no benefit from statins [43]. It should also be noted that CACS showed an additive role to perfusion PET for patient risk stratification [44, 45]. As such, the additional acquisition of a dedicated CT to calculate CACS should be advised in patients without known CAD in view of the diagnostic and prognostic value of CACS [41, 46–48].

Finally, as third indication, CCTA can complement information from MPI, in particular in patients with unclear findings on their perfusion scan, thereby informing on the need for invasive coronary angiography (ICA).

As hybrid PET/CT imaging will increase the radiation dose to the patient, since both techniques utilize ionizing radiation, additional exposure to radiation caused by the use of two instead of one imaging modality needs to be weighed against potential benefits, especially in young individuals.

Integration of the PET and CT information and image fusion enable effective combination of myocardial perfusion imaging with anatomical coronary plaque assessments. Several studies support the complementary role of the diagnostic and prognostic information provided by CCTA and PET perfusion imaging in the evaluation of CAD. For example, the capability of PET perfusion imaging to detect myocardial ischemia [49, 50] can be useful in further assessment of intermediate angiographic stenoses detected by CCTA [51]. Assessment of ischemia by perfusion imaging can be also helpful in further evaluation of coronary segments with image artefacts on CCTA [51]. The use of hybrid imaging may be particularly helpful in evaluation of patients with previous revascularization in whom there may be artefacts from metal objects such as coronary stents [52] or after coronary artery bypass graft surgery (CABG). The Bypass CTCA Trial showed that CCTA before invasive coronary angiography (ICA) leads to reductions in procedure time [53]. Coupled with MPI, this imaging modality would provide important information on location, extent, morphological characteristics and hemodynamic significance of coronary lesions, thus allowing for a more streamlined decision on the most adequate therapeutic options. Furthermore, a hybrid approach can have important advantages in selected patients, e.g. those with unclear MPI or those with a need to correctly assign an ischemic area to the correct subtending territory. In fact, regional perfusion defects within the myocardium are usually assigned to the relevant vascular territories by applying a mental co-registration between MPI and standardized LV segmentation models [54] but coronary anatomy can vary considerably among individuals [55], and the correct assignment can be challenging in case of ischemia e.g. in the standard right coronary artery (RCA) and left circumflex artery (LCX) territories [56]. As such, the use of hybrid MPI/CCTA imaging may be advised in order to assess the hemodynamic significance of residual stenoses in patients who had undergone PCI in another vessel, and in those patients with multiple potentially obstructive stenoses on a previously performed CCTA [57]. Finally, with regard to myocardial flow quantification, hybrid imaging can help to differentiate reduced myocardial flow reserve due to multi-vessel CAD from coronary microvascular dysfunction in the absence of obstructive CAD [58].

Consistent with these advantages over stand-alone modalities, several studies have shown that combination of CCTA with the detection of myocardial ischemia by PET perfusion imaging is feasible and provides high diagnostic accuracy for the detection of obstructive CAD [10, 59–62].

Furthermore, a meta-analysis compared hybrid CCTA and perfusion imaging with either SPECT, PET or MRI with CCTA alone (12 diagnostic studies and 951 patients in total) for the detection of obstructive CAD defined as luminal diameter reduction of at least 50% by invasive angiography [63]. This meta-analysis found that whilst the pooled sensitivity of hybrid imaging was comparable to that of CCTA on per-patient (91% vs. 90%) and per-vessel (84% vs. 89%) basis, the specificity was markedly improved at both the per-patient (93% vs. 66%) and per-vessel (95% vs. 83%) levels. The overall diagnostic performance was also improved on a per-vessel basis (area under the curve 0.97 vs. 0.92).

A more recent single-center prospective study compared hybrid imaging with stand-alone imaging in 208 patients who underwent CCTA, [^15^O]-Water PET perfusion imaging and ICA combined with measurement of fractional flow reserve (FFR) in all arteries [64]. In this study, the addition of functional imaging to CCTA improved specificity for the detection of obstructive coronary artery disease, at expenses of reduced sensitivity. Another recent study also found that in patients with suspected obstructive stenosis at CCTA, [^82^Rb]Chloride PET increased specificity up to 89%, which renders [^82^Rb]Chloride PET an important second-line imaging technique after CCTA. It should be noted that the loss in sensitivity may be due to the use of 0.80 as a threshold for FFR, given that a clear threshold for FFR is challenging to determine [65].

More importantly, emerging evidence indicates that combined anatomic and functional information can play a complementary role by further refining cardiovascular risk prediction. In patients with obstructive lesions on CCTA, additional evidence of ischemia on hybrid imaging is associated with a high event risk [52].

The association of SPECT and CCTA results in improved diagnostic accuracy over each imaging modality as stand-alone [7, 9, 10]. In this regard, the addition of CT also allows the detection of non-obstructive CAD, also providing with prognostic information. However, routine acquisition of combined SPECT/CCTA imaging is currently not advised owing to the higher radiation exposure compared to MPI alone. Conversely, a more important role may be secured for hybrid PET/CCTA. The clinical indications may be similar to those of perfusion PET scans [14, 66, 67], but in the abovementioned clinical scenarios a gain in diagnostic and prognostic values is expected.

The evidence regarding the use of SPECT/CT and PET/CT systems in Equilibrium Radionuclide Angiocardiography (ERNA) is scarce. Recently, Carsuzaa et al. investigated the use of a 3D-ring CZT (SPECT-CT) general purpose system for left ventricular ejection fraction (LVEF) and cardiac function evaluation [68]. Based on their preliminary results, there was no significant difference in LV volumes, LVEF and right ventricular ejection fraction (RVEF) between this system and a cardiac-dedicated CZT camera. Moreover, Ben Bouallègue et al. showed that gated first-pass [^18^F]FDG PET/CT of the cardiac cavities may represent an alternative technique for LV function evaluation [69]. It should be noted that the clinical use of ERNA is now declining and often used as second line imaging test after echocardiography techniques and CMR, which are not associated with radiation exposure.

#### Pitfalls and artefacts

**Fig. 2:**
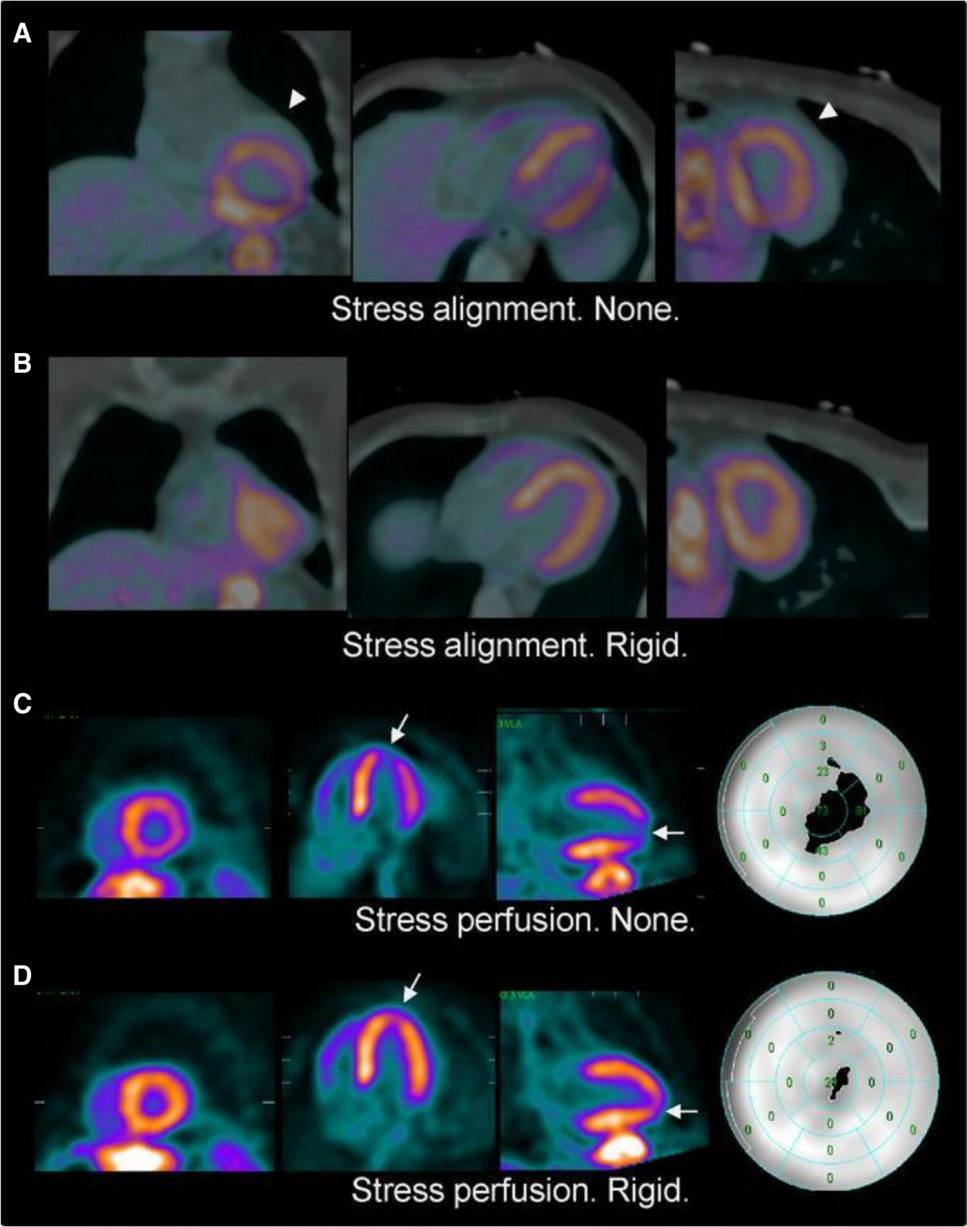
False-positive nuclear scan due to the CT registration error resolved by the automatic correction in a 44-year-old male presenting with shortness of breath. PET and CTAC images fused before alignment (**A**) and after rigid alignment (**B**). Misalignment is indicated by the white arrow head (A). Stress perfusion PET images before alignment (**C**) and after alignment (**D**) and polar maps are shown with the white arrow indicating the perfusion artifact due to misalignment. Quantitative total perfusion defect (TPD) was 11% (C right), when no correction for alignment was performed (ITPD after manual verification was also 11%) compared to a normal scan (TPD 1.8%) after automatic registration (D right). Invasive coronary angiography did not show significant coronary artery disease (Reprinted with Permission of Springer from [71], no changes were made)

AC is intrinsically more accurate for PET than for SPECT acquisitions owing to emission of simultaneous dual vs. single gamma rays, the higher energy of the emitted photons and the more efficient detection of signal. The attenuation map is typically based on a transmission scan like CT, and more often relies on measured transmission data acquired before (preinjection), during (simultaneous), or after (postinjection) the emission scan. This approach is used both for PET and SPECT, taking into consideration the different contribution of photon attenuation in the two modalities. Due to the different physical principles, the magnitude of the correction factors required for PET is far greater than for SPECT [70]. Consequently, precise co-registration between MPI and CT is required to yield accurate generation of the attenuation map. Conversely, incorrect co-registration between MPI and CT can result in apparent perfusion defects due to errors in the generation of the attenuation map (Fig. 2) [71]. For the same reason, some perfusion abnormalities may falsely disappear on AC-images, thus reducing the sensitivity of the examination [25, 72, 73].

The use of CT for tissue AC in MPI therefore requires accurate analysis of the images acquired with the two techniques to verify the correct superposition of the images and training in the interpretation of the corrected SPECT images to identify the presence of artefacts induced by the attenuation correction.

### Inflammation and infection imaging

The number of patients referred for [^18^F]FDG PET/CT imaging to depict cardiovascular inflammation or infection has continuously increased over the past 20 years. For these clinical indications, CT protocols allow for accurate AC of PET acquisitions, but can also provide anatomical information to aid in the diagnosis.

#### Endocarditis

[^18^F]FDG PET/CT has a recognised role in the diagnosis of infective endocarditis (IE), mainly in patients with prosthetic heart valves (PHV) or cardiac implantable electronic devices (CIED). The European Society of Cardiology (ESC) guidelines for the management of IE, published in 2023, recommend the use of additional imaging modalities when echocardiography and blood cultures result in a “possible” diagnosis of IE or a “rejected” diagnosis with persisting high suspicion [74]. Hence, CT and PET scans now have a central role (as does echo) in diagnosing valvular, perivalvular/periprosthetic and foreign material anatomic and metabolic lesions [75, 76]. The international consensus document on CIED Infection criteria [34] as well the European Heart Rhythm Association (EHRA) of the ESC international consensus document on cardiac implantable electronic device infections [77] also introduced nuclear imaging for the diagnosis of CIED-related infections and complications. In this clinical scenario, CT is used for AC and for anatomical localization of both cardiac and extracardiac foci of [^18^F]FDG uptake and to evaluate their association with the implanted material. The combined acquisition of [^18^F]FDG PET with CCTA within one single imaging session yields more accurate localisation of the [^18^F]FDG PET signal in relation to cardiac structures and, in the case of prosthetic valve endocarditis, also aids in the detection of paravalvular abscesses and pseudoaneurysms. Hence, hybrid PET/CCTA allows to combine the high sensitivity of [^18^F]FDG PET to detect infectious foci with the high spatial resolution of CCTA to detect structural lesions associated with IE. As such, [^18^F]FDG PET/CCTA may allow simultaneous assessment of two major Duke criteria (abnormal activity around the site of prosthetic valve implantation by [^18^F]FDG PET/CT and definite paravalvular lesions by CCTA, [78] in a single study. Additionally, minor Duke criteria (vascular phenomena such as major arterial emboli, septic pulmonary infarcts, infectious aneurysm) can be confirmed with whole-body acquisitions. Finally, the simultaneous acquisition of CCTA can also assess the coronary arteries and thoracic aorta in candidates for invasive procedures, thereby guiding decision-making.

**Fig. 3:**
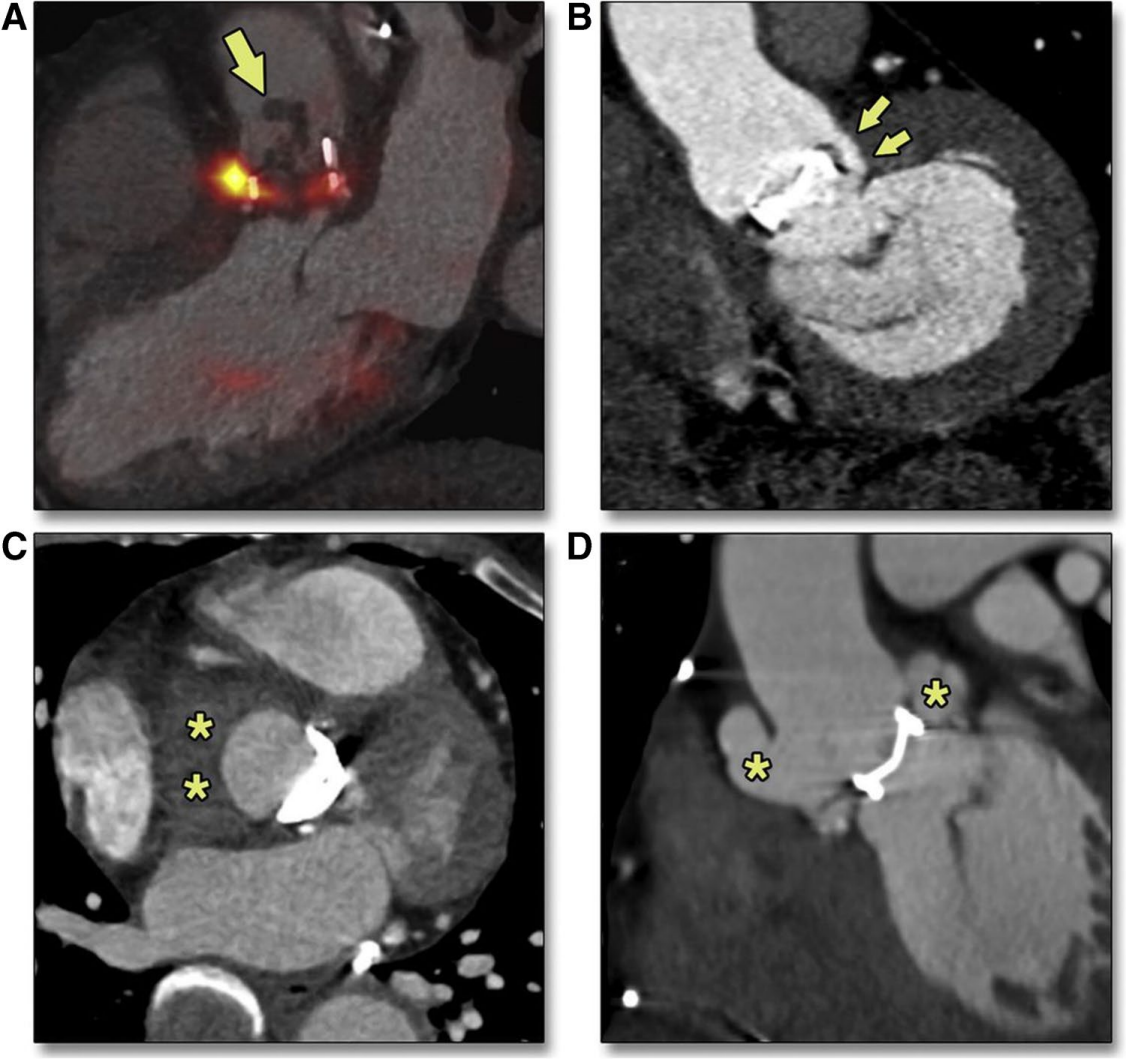
**A** Large aortic vegetation (arrow) in late (3 years post-implantation) prosthetic aortic valve endocarditis, blood culture–positive for Staphylococcus epidermidis. (**B**) Paravalvular fistula (arrows) in late (15 years post-implantation) prosthetic aortic valve endocarditis, culture-positive for Streptococcus bovis. (**C**) Poorly delimited perivalvular soft tissue mass (asterisks) corresponding to a periprosthetic abscess in a patient with infective endocarditis (9 years post-implantation), culture-positive for Enterococcus faecalis. (**D**) Multilobulated periprosthetic pseudoaneurysm (asterisks) in a patient with relapsing endocarditis (11 months post-implantation), culture-positive for Mycobacterium avium intracellulare. CTA = computed tomography angiography. Reprinted with permission of Elsevier from [79]

Studies exploring the additional value of combining CCTA with [^18^F]FDG PET in IE are scarce, but there is a clear tendency toward the demonstration of an improvement in diagnostic accuracy if the two techniques are combined (Fig. 3) [79]. PET/CCTA allows for reducing the rate of equivocal examinations compared to PET with standard contrast-enhanced CT, mainly due to the correct attribution of focal [^18^F]FDG uptakes to the prosthetic material especially in cases of incomplete myocardial suppression. Furthermore, the more accurate co-registration between PET and CCTA and the detection of a significantly higher number of structural lesions associated with IE compared with echocardiography or standard [^18^F]FDG PET/CT allows for improved diagnostic accuracy. In a study by Pizzi MN et al. [80], the addition of CCTA to the standard [^18^F]FDG PET/CT protocol reduced the number of equivocal IE cases from 20 to 8%, mainly by reclassifying doubtful cases into negative ones. This translated into higher diagnostic accuracy for [^18^F]FDG PET/CCTA than standard [^18^F]FDG PET/CT with a sensitivity, specificity, PPV, and NPV of 91%, 901%, 93%, and 88%, vs. 86%, 88%, 90%, and 83%, respectively.

The presence of these structural lesions as well as information on the size, anatomy and calcification of the aortic valve, root, and ascending aorta in patients with aortic IE and the presence of coronary artery disease may be relevant for subsequent clinical and surgical decision-making. Recent studies demonstrated that [^18^F]FDG PET/CT combined with CCTA can be useful after transcatheter aortic valve replacement (TAVR). Salaun E et al. reported that a multimodality imaging approach, according to the 2015 ESC modified criteria, has an excellent diagnostic value in patients with suspected IE after TAVR (sensitivity = 100% for definite-IE diagnosis) compared to the modified Duke criteria (sensitivity = 50%) [81]. Similar advances have been reported in post-operative patients after TAVR [82] as well as in patients with congenital heart disease and intravascular or intracardiac prosthetic material [83]. In CIED infections, the addition of CCTA may have limited additional value, but further studies are needed to evaluate its role in this setting.

White-blood-cell (WBC) scintigraphy has demonstrated its value for the diagnosis of IE [84]. WBC scintigraphy provides high specificity for the detection of prosthetic or CIED infection and may help to discriminate inflammation from infection in the presence of doubtful [^18^F]FDG PET images [85]. For WBC scintigraphy, planar acquisitions are followed by SPECT acquisitions to increase the sensitivity of detecting the accumulation of radiolabelled leukocytes in the cardiac region. SPECT acquisitions are usually associated with non-ECG-gated low-dose CT acquisitions for attenuation correction and for a more precise localisation of the signal to the heart. Considering the strengths and weaknesses of [^18^F]FDG PET (higher sensitivity, lower specificity) and WBC SPECT/CT (lower sensitivity, higher specificity) [86], it is advised that [^18^F]FDG PET is performed first with WBC scintigraphy used as second line test in case of unclear finding of [^18^F]FDG PET imaging.

Pitfalls and artifacts

Similar to perfusion imaging, spatial mismatch may exist between PET or SPECT and CT images, as PET or SPECT acquisitions are averaged over few minutes, whereas CT acquisitions last only few seconds. Integration of the all the clinical data plays a crucial role to avoid misinterpretation, this includes the details of the surgical technique, the type of material implanted (e.g. use of surgical glue which may cause false positive [^18^F]FDG uptake) as well as the presence/absence of associated anatomical lesions on CT and the accurate localization of [^18^F]FDG uptake to prosthetic material.

Typical pitfalls of hybrid PET/CCTA imaging in the diagnostic workup of endocarditis are:

- *inadequate suppression of physiological myocardial [*^*18*^*F]FDG uptake*. Fusion with CCTA may improve the delineation of the prosthetic valve and allow for better differentiation of pathological periprosthetic [^18^F]FDG uptake from residual myocardial FDG uptake. However, a specific patient’s preparation is also mandatory (carbohydrate-free diet for 24–72 h prior to the examination and/or administration of heparin [87].
- *patient movement.* It may result in emission-transmission misregistration, but many software programs now allow proper realignment and co-registration of the images.
- *CT artefacts.* For example, metallic structures and highly dense calcium have high attenuation coefficients, which lead to local overestimation of [^18^F]FDG activity or WBC signal and may result in false-positive findings. For the interpretation of [^18^F]FDG PET/CT and WBC-SPECT acquisitions in patients with a suspicion of infected prosthetic material, current recommendations have therefore underscored the importance of confirming the presence of an abnormal [^18^F]FDG or WBC signal on the non-attenuation corrected PET or SPECT images which increases the likelihood of pathological vs. artefactual findings.
- Biologic and foreign material commonly results in positive [^18^F]FDG uptake. Especially the aortic prostheses may have persistent, high signal at the anastomosis sites. CT allows the assessment of periannular aortic epicardial fat density, which may help demonstrating true infectious foci.
- *Antibiotic therapy*. [^18^F]FDG PET imaging once patients have been established on antibiotic therapy may lead to false negative results particularly in patients in whom the C-reactive protein levels have been effectively suppressed.
- *Postoperative assessment*. The assessment of IE affecting prosthetic valves in the postoperative period is more challenging due to high heterogeneous signal persistent in uncorrected images, sometimes associated with morphological lesions on CT scan, which are para-physiological but may mimic an IE.

#### Vasculitis

Large vessel vasculitis (LVV) is the most common form of primary vasculitis and encompasses giant cell arteritis (GCA) and Takayasu’s arteritis (TA) [88, 89]. Typical aspects in favor of active LVV on CTA or contrast-enhanced MRI are the presence of diffuse, non-calcified circumferential thickening (> 2 mm) of the vascular wall with adventitial enhancement. CTA can identify stenotic lesions, especially in TA [90] and allows for the evaluation of both the location and extent of the lesions with excellent spatial resolution [91]. Moreover, CTA helps to detect morphological alterations, such as vascular stenosis, occlusion, or ectasia, as well as surrounding edema or tissue reactions. In chronic disease stages, CTA is an alternative to MRI for detecting late complications such as aneurysm formation, which may affect 1/3 of patients after long-term follow-up [92] and is helpful in planning percutaneous and surgical treatment [93]. In addition to morphological imaging, [^18^F]FDG PET provides high sensitivity for the detection of inflammation affecting large arterial walls [94, 95]. Concerning specificity, CTA has a reported value of 85%, while [^18^F]FDG PET as high as 100% for the detection of GCA-related aortitis [96]. As such, a hybrid PET/CTA approach is useful to combine detection of the morphologic and metabolic features that characterize LVV. On CTA, wall thickening may be mistaken for atherosclerotic plaques and focal [^18^F]FDG uptake may be detected in inflamed atherosclerotic plaques on PET [97]. Combined analysis of morphological aspects of the vessel wall with CTA and the [^18^F]FDG signal on PET may help define whether the vascular [^18^F]FDG signal originates from active vasculitis or from inflammatory activity in atherosclerotic plaques. Again, LVV is usually associated with a circumferential pattern of increased [^18^F]FDG PET activity and wall thickening that can be differentiated from the more patchy distribution of plaque and [^18^F]FDG uptake associated with atheroma. Evidence is limited for the role of MRI in GCA, but MRI is widely used to assess cranial involvement in GCA and to image the morphological vascular changes observed in TAs. Patients’ younger age at diagnosis and need for lifelong surveillance imaging may favor the use of MRI in combination with PET, in order to reduce radiation burden in patients with Takayasu’s arteritis, although there is currently limited evidence to support the use of hybrid imaging in this context.

Pitfalls and artifacts

CTA:

- motion artefacts in proximal segments of the thoracic aorta on non-gated CT acquisitions. This can be easily overcome by performing ECG-gated acquisitions of the thoracic aorta.
- The use of contrast agent may reduce the accuracy of attenuation correction, unless CT data are collected in the equilibrium or venous phase (i.e. delayed acquisition), with the advantage of radiation dose reduction.

[^18^F]FDG PET:

- Patient movement occurring between PET and CT acquisitions, in particular for the imaging of supra-aortic trunks. To minimize this consider using a head-neck holder and attempt to shorten the delay between CT and PET acquisitions (dedicated PET/CT step).
- lower sensitivity of [^18^F]FDG PET for active vasculitis in presence of residual blood activity; consider later acquisition time points after acquisitions for vascular imaging [98].
- Impact of glucocorticoids (GC) treatment on the intensity of the [^18^F]FDG signal. Imaging should be performed as early as possible, but should not delay, the initiation of GC treatment. [99]. It should be noted that a recent study showed that the impact on diagnostic accuracy is negligible for patients with up to 3 days of ongoing GC therapy, and is significantly affected only after 10 days of therapy [99].

#### Sarcoidosis

Sarcoidosis is a multisystemic granulomatous disease of unknown etiology, with cardiac involvement in an estimated 20–30% of patients [100, 101]. While whole-body [^18^F]FDG PET imaging allows identification of active sarcoid granuloma across the body with high sensitivity, histopathological confirmation is needed. Extra-cardiac biopsy should be preferred to confirm the diagnosis because endomyocardial biopsy is associated with a low diagnostic yield and a peri-procedural risk. In this context, [^18^F]FDG PET imaging is useful to identify the most accessible lesion showing an inflammatory suspicious pattern that has the highest probability of confirming the diagnosis on histology [102]. PET/CT is the first-line hybrid imaging approach in patients with a suspicion of cardiac sarcoid as PET/CT systems are more accessible than PET/MRI and more suitable for whole-body imaging. Patients referred with a suspicion of sarcoidosis should be prepared with a low-carb high-fat diet 24 h prior to imaging to suppress the physiological [^18^F]FDG uptake in the myocardium as previously reported [87]. Patient preparation is crucial to avoid misinterpretation of imaging findings related to inaccurate suppression protocol rather than to pathological tracer accumulation [103, 104]. This allows for the detection of active granulomas in the heart in addition to extracardiac disease. CT acquisitions associated to PET/CT usually consist of a non-gated low-dose CT to correct PET images from attenuation and identify the anatomical structures with high degree of [^18^F]FDG uptake. In the cardiac region, care should be taken to discriminate [^18^F]FDG uptake localized in the heart from adjacent mediastinal lymph nodes or lung parenchyma. In patients with high suspicion of cardiac sarcoidosis, the addition of rest MPI (if available) to cardiac [^18^F]FDG PET acquisitions is advised to increase the specificity of image analysis and improve risk stratification and to predict the likelihood of recovery from atrioventricular block in advanced disease stages [105, 106]. To note, CAD can also mimic a similar perfusion/metabolism pattern, and this condition should be ruled out. Alternatively, comparison can be made with CMR late gadolinium enhancement (LGE) images to ensure areas of increased [^18^F]FDG activity line up with regions of myocardial injury on the CMR. The acquisition of ECG-gated or contrast-enhanced CT acquisitions is not advised in this clinical indication. Reduction of radiation burden due to CT is particularly important given the predominantly young age of patients who often undergo repeated [^18^F]FDG PET acquisitions to monitor the efficacy of immunosuppressive treatments.

#### Cardiac amyloidosis (SPECT-CT)

**Fig. 4:**
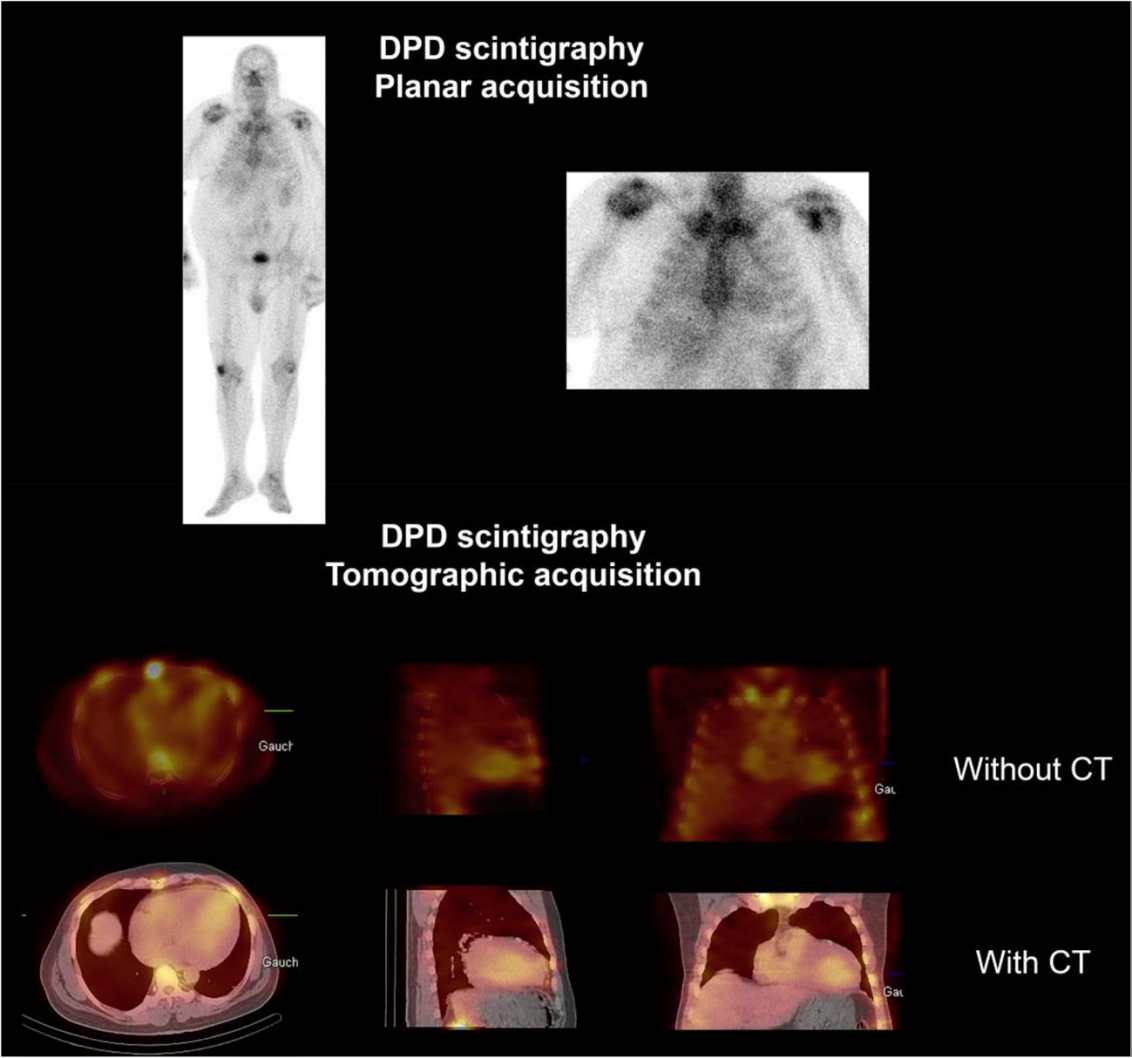
False-positive cardiac uptake using bone scintigraphy with blood pool residual activity. Planar acquisition was scored with grade 1 according to Perugini classification. SPECT acquisition fused with low-dose CT shows that the signal is located in left ventricular cavity, without bone tracer uptake within the myocardium

Cardiac scintigraphy with bone-avid tracers plays an important role in the diagnosis of transthyretin-related (ATTR) cardiac amyloidosis (CA) [107]. The binding of bone-avid tracers can be detected on planar acquisitions. However, SPECT acquisitions centered on the heart, with additional low-dose CT, are advised after planar acquisitions, particularly in cases of weak or doubtful cardiac uptake of the bone tracer to differentiate between myocardial uptake and blood pool activity preventing potential false positive examinations [108, 109] (Fig. 4). Furthermore, AC SPECT acquisitions may prove useful for signal quantification of cardiac uptake of bone tracers and to monitor the impact of new treatments on amyloid load [110].

### Summary of clinical scenarios. Clinical consensus statement by EANM and EACVI

Different clinical scenarios are summarized in Table 2, along with suggested PET/CT protocols for each indication. A low-dose CT for anatomical localization and AC should be routinely used for all the indications listed. In the assessment of myocardial perfusion, a dedicated CT acquisition for CACS in patients without previous stenting or CABG is useful to improve risk stratification of patients in association to the results of MPI. Finally, the use of hybrid PET/CCTA may provide additional information for the myocardial perfusion imaging and the evaluation of patients with a suspicion of endocarditis.

**Table 2:** Summary of the proposed CT protocols for combined PET/CT imaging across different clinical indications

Imaging modality	Clinical indication	CT for attenuation correction	Nonenhanced CT for anatomical localization	ECG-gated nonenhanced CT (CACS)	CT angiography	ECG-gated CT angiography
SPECT-CT	**MPI**	required	required	required	non-appropriate	possible
	**Amyloidosis**	adequate	required	non-appropriate	non-appropriate	non-appropriate
	**Endocarditis**	required	required	non-appropriate	non-appropriate	possible
PET-CT	**MPI**	required	required	required	non-appropriate	adequate
	**Viability**	required	required	adequate	non-appropriate	adequate
	**Endocarditis**	required	required	non-appropriate	adequate	adequate
	**Sarcoidosis**	required	required	non-appropriate	non-appropriate	non-appropriate
	**Vasculitis**	required	required	non-appropriate	adequate	possible
	**Atherosclerosis**	required	required	adequate	adequate	possible

## Hybrid imaging with MR

### MR protocols

While hybrid PET/MR scanners are currently not widely deployed in radiologic centers, this approach still holds potential for the assessment of various cardiac diseases [111]. PET/MR combines the high sensitivity of PET imaging for the detection of tracers with the precise assessment of cardiac function and tissue characterization offered by multiparametric MR. This hybrid modality therefore holds particular value when assessing cardiovascular structures such as the myocardium or the vascular wall, for which potential advantages over CT can be envisioned [112–114].

Standard cardiac MR sequences used to image these structures can be acquired simultaneously with the PET acquisition. Another key advantage of PET/MR is the lack of radiation associated with MRI, which is particularly relevant when imaging young patients or if repeated imaging is required for monitoring disease activity [115].

An important consideration in PET/MR imaging is AC. AC is relatively simple when performed using CT acquisitions as both imaging techniques are based on the diffusion of high-energy photons through tissues. However, AC of PET images with MR is less straight forward, and requires extrapolation of tissue attenuation maps after identification of keys attenuating structures on dedicated MR sequences. Recently, some important limitations with vendor-specific attenuation correction PET/MR sequences have however been emphasized when applied to cardiovascular disease states. In particular, breath-held AC sequences lead to the generation of artifacts in the PET data (which by contrast is acquired throughout the respiratory cycle) at the lung-heart and lung-diaphragm borders. Moreover, high signal artifact is often generated within the trachea and bronchi which are not well segmented with these sequences. Both these forms of artifact can make interpretation of PET data in and around cardiac or vascular structures challenging [116]. Bespoke free breathing radial vibe AC sequences have been developed for cardiovascular applications that overcome these limitations [116]. In particular, these sequences do not generate the artifacts described here-above and therefore allow for a more reliable interpretation of cardiovascular PET data.

Moreover, it should be noted that misalignment of MR and PET datasets may occur during stress MPI on a hybrid PET/MR scanner and myocardial creep may substantially account for this [117]. Methods have been developed, which yield higher accuracy in the PET/MR coregistration in cardiovascular imaging [118], but to-date a post-processing adjustment of PET and MR data should be taken into account.

Further work is still required to explore machine learning approaches that may generate CT-like AC maps from the MRI data [119].

### Cardiac imaging

#### Viability

Repetitive stunning or chronic hypoperfusion may affect the myocardium and result in wall motion abnormalities, a state called “hibernating myocardium”. In this state, the affected myocardium is still vital, and may recover its function following revascularization [120, 121]. Hypoxia and ischemia cause a shift in myocardial substrate metabolism from free fatty acid oxidation toward glucose consumption, which can be identified by the preserved or even increased glucose metabolism in hibernating myocardium. Of the various viability imaging modalities, [^18^F]FDG PET and LGE MR imaging are the most commonly used.

**Fig. 5:**
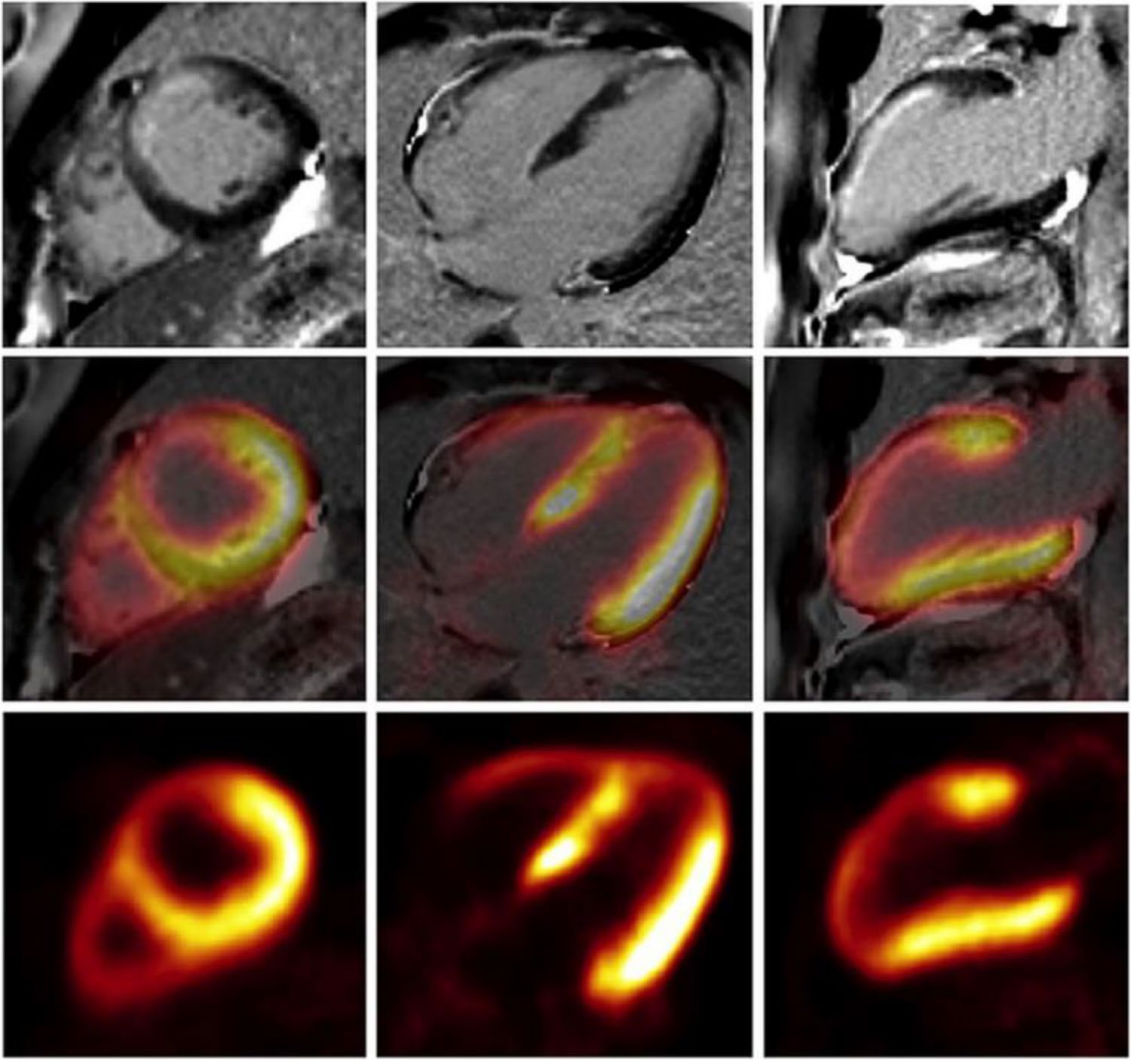
Viability imaging using FDG PET and LGE MRI in a patient with a scar of the anterior wall. Reduced glucose metabolism and late gadolinium enhancement is present in the area of scarring in the anterior wall (reprinted with permission from [161])

While a hybrid PET/MR imaging approach is expected to increase diagnostic accuracy by combining the strengths of both modalities, its real impact in clinical practice still needs to be established. Most of the few existing studies to date, describe substantial agreement between the two approaches [122, 123]. Further, a small study of patients with chronic total coronary occlusions demonstrated that a combined approach may allow better prediction of left ventricular wall motion recovery after revascularization [124]. Figure 5 shows one example of integrated PET/MRI viability imaging.

#### Inflammation and infection

**Fig. 6:**
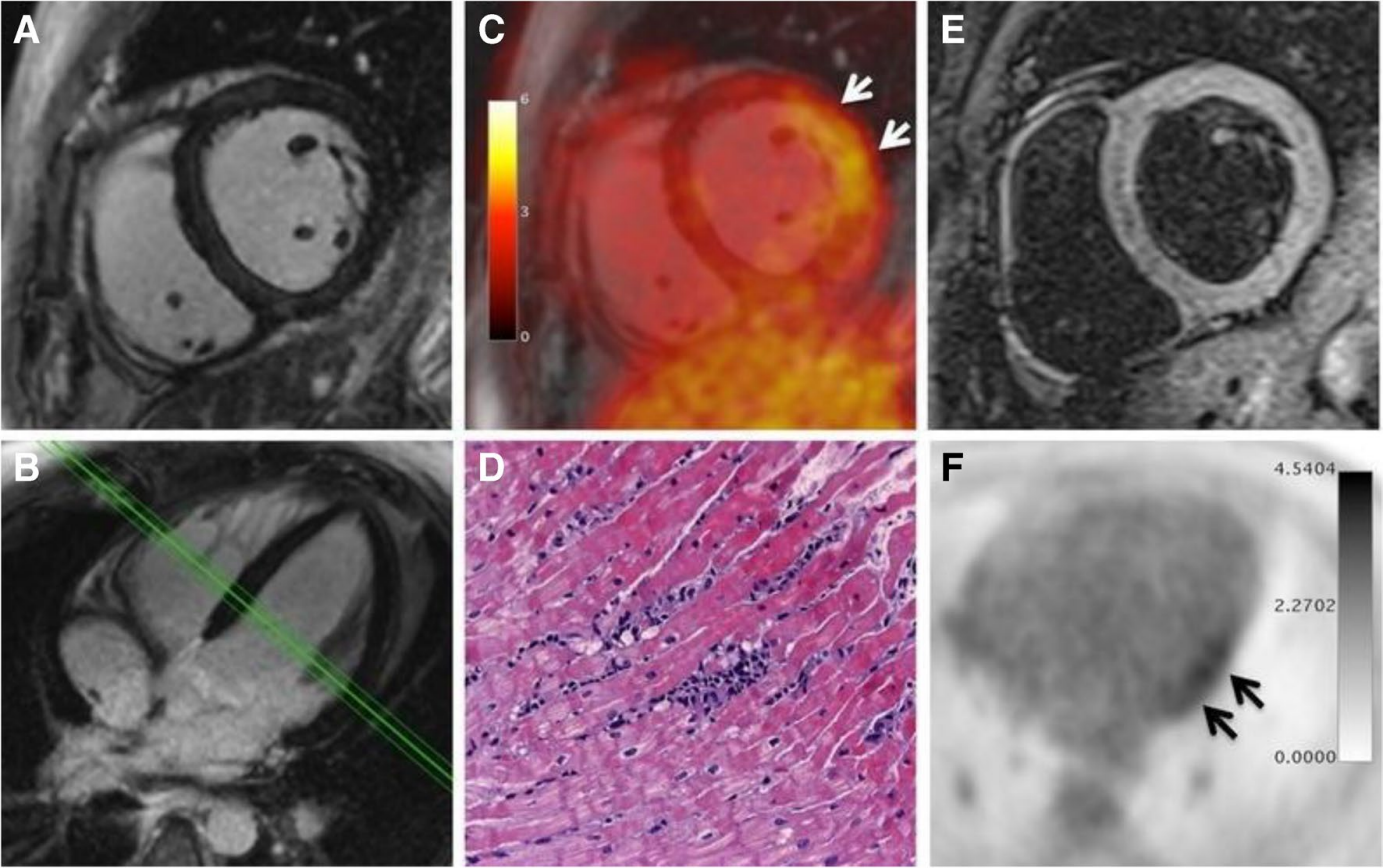
PET/MRI of a 30-year-old male patient with myocarditis. Despite LGE images (**A**, **B**) show no abnormality, PET images (**C**, **F**) demonstrate focal FDG uptake in the lateral wall. T2-weighted imaging (**E**) show mild myocardial edema. Diagnosis of borderline myocarditis was confirmed by histopathological assessment after endomyocardial biopsy demonstrating sparse inflammatory infiltrates but no myocardial necrosis (**D**). Reused with permission from [130]

MR has limited value in endocarditis or CIED. However, it does provide useful information in myocarditis [125]. Myocardial inflammation and fibrosis can be assessed using LGE or T1/T2 mapping techniques (ECV) and MRI is also the modality of choice to identify pericardial effusions and wall motion abnormalities. On the other side, [^18^F]FDG PET offers high sensitivity for the detection of active myocardial inflammation, and differentiation from chronic burnt out disease states, thereby providing complementary information to cardiac MRI. Taken together, there is a clear rationale to support consideration of hybrid PET/MR imaging in the assessment of myocarditis, in view of its potential to increase in diagnostic accuracy for the detection of active myocardial inflammation [126]. After initial positive case reports on the use of hybrid [^18^F]FDG PET/MR imaging for the diagnosis, grading, and monitoring of myocarditis using [^18^F]FDG PET/MRI [127–129], a first prospective study of 65 patients on this topic was published [130]. Compared with a variant of the 'Lake Louise Criteria' as the reference standard, [^18^F]FDG PET had a sensitivity of 74% and a specificity of 97%. However, the most significant observation was that patients with biopsy-proven myocarditis may demonstrate pathologic myocardial [^18^F]FDG uptake without abnormalities on MRI, possibly representing an early stage of the disease when no morphologically detectable cardiac changes are yet present. Figure 6 demonstrates a case example of [^18^F]FDG PET/MR in myocarditis.

Finally, some reports show that hybrid PET/MR imaging may identify inflammation and microcalcification activity in the carotid and coronary arteries [116]. However, technical improvements are needed before a wide clinical application is feasible.

#### Myocardial masses

Cardiac masses are a diagnostic challenge requiring not only morphological identification and classification but also distinction between benign and malignant lesions [131, 132]. To date, cardiac MR imaging represents the most accurate imaging approach for cardiac mass assessment owing to its excellent spatial resolution and ability in tissue characterization [133–135], [^18^F]FDG PET may provide complementary information, helping to discriminate malignant from benign lesions and allowing the staging of malignant tumours.

[^18^F]FDG maximum standardized uptake (SUVmax) is higher in malignant vs. benign cardiac masses [136, 137]. The prevalence of malignant tumors is high among cardiac masses with SUV max > 5.0 on [^18^F]FDG PET, but the interpretation of PET acquisitions should be combined with other imaging modalities and the clinical context as false-positive benign lesions may occur [132, 138]. The incremental value of PET/MR hybrid imaging has been investigated by Nensa et al., who demonstrated an important role for this technique in differentiating between scar tissue and cardiac tumour relapse after surgery [139]. Aghayev et al. [140] showed that CMR has higher sensitivity (98%), while [^18^F]FDG PET yields high specificity (84%) in differentiating benign from malignant masses, and the integration of the information coming from both modalities yields 85% sensitivity and 88% specificity in diagnosing malignant masses. These data suggest that [^18^F]FDG PET/MR provides improved characterization of cardiac masses compared to stand-alone PET and MR modalities [141].

#### Cardiac amyloidosis

**Fig. 7:**
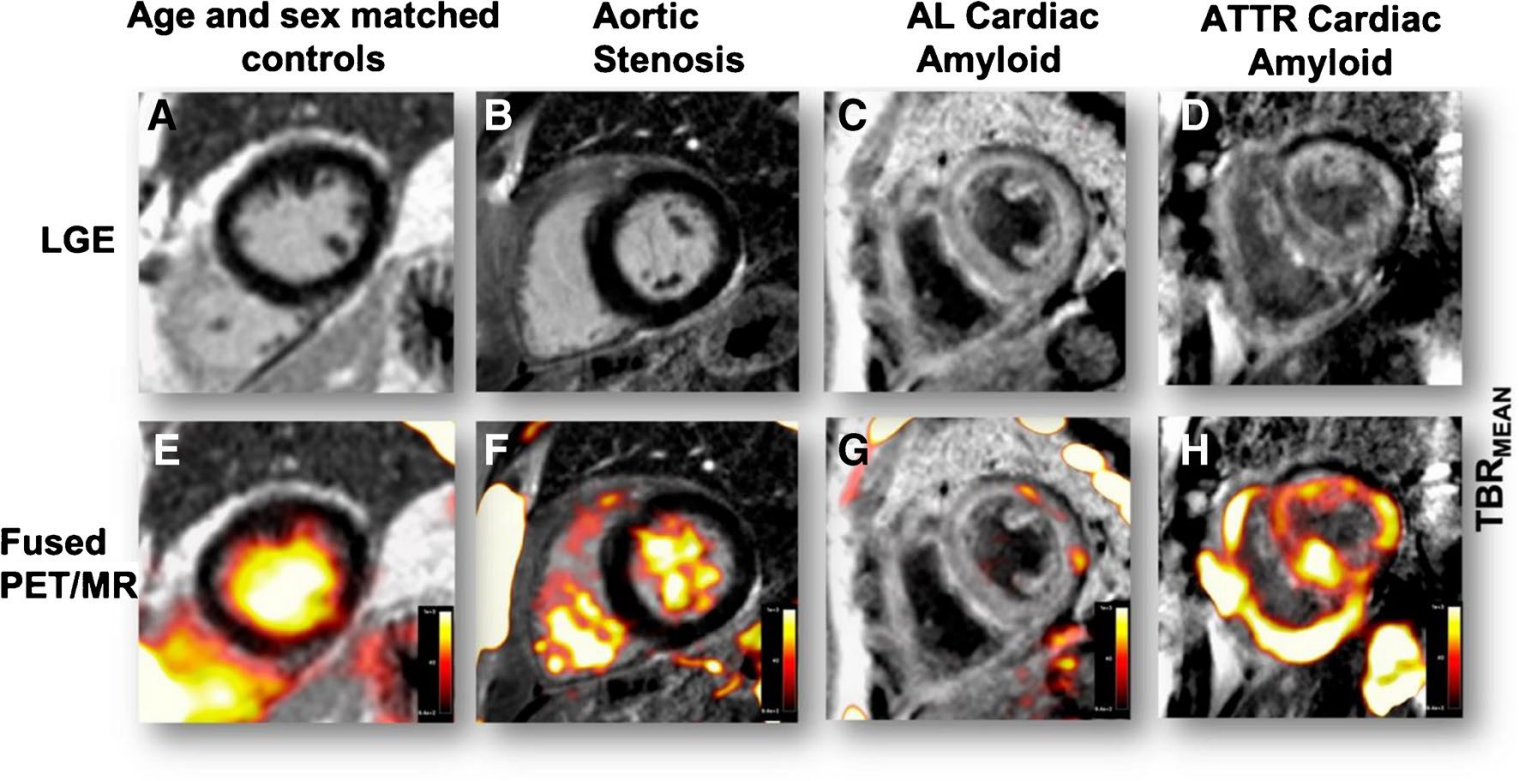
Patterns of [^18^F]fluoride uptake among different patients’ cohorts. **A**: delayed enhanced image of a control subject with normal myocardial mass and no LGE. The corresponding fused PET/MR image (E) shows uptake only in the blood pool. **B**: patient with aortic stenosis and elevated LV mass. Similar to healthy controls, no [^18^F]fluoride uptake is evident (**F**). Uptake is greater in the blood pool than myocardium. **C**: patient with AL amyloidosis displaying the characteristic myocardial nulling difficulties with LGE found in cardiac amyloidosis. **G**: patchy lateral wall uptake greater than the blood pool. **D**: similar LGE findings, but this time in ATTR CA. **H**: intensive biventricular uptake in a patient with ATTR CA. Reprinted with permission of Springer Verlag from [142]. No changes were made

Several papers investigated the potential role of hybrid PET/MR imaging in the evaluation of cardiac amyloidosis (CA). In view of its similarity to the already validated diphosphonate scintigraphy, [^18^F]fluoride was investigated in a pilot study in 2016, which demonstrated increased [^18^F]fluoride activity in subjects with ATTR CA compared to both healthy individuals and those with AL amyloidosis [112]. Interestingly, the increased uptake correlated well with areas of LGE in MR imaging. A recent multicenter study also confirmed [^18^F]fluoride PET/MR’s ability to differentiate between AL and ATTR subtypes of CA. (Fig. 7) [142]. The multicentre I-CARE study is investigating whether the improved quantification offered by PET can be used to track changes in ATTR disease burden with time and in response to therapy (**NCT05776212**). However, some important limitations should be kept on mind. Due to the low uptake of [^18^F]fluoride in the normal myocardium (which is usually lower than uptake in the blood pool with TBR values of 0.5–0.7) even quite marked increases in myocardial tracer uptake can be quite hard to visualize. For example, even a 100% increase in myocardial tracer uptake might look similar in uptake to blood pool and requires quantification to be appreciated fully [143, 144].

Several other radiopharmaceuticals for PET imaging have been developed for the detection of amyloid protein accumulation in the brain in Alzheimer's disease. Even though amyloid proteins accumulating in the brain and in the heart differ (amyloid-beta precursor protein vs. TTR or light chains, respectively), they have in common the presence of beta-sheet structures. Hence, several radiopharmaceuticals initially dedicated to the detection of Alzheimer's disease have been evaluated for the diagnosis of CAs. Baratto et al. showed that [^18^F]Florbetaben PET/MRI can effectively localize systemic amyloid deposition [145]. Similar results were reported using [^11^C]PIB PET/MR by Bi et al., wherein 23 Patients were investigated (13 diagnosed with CA and 10 negative controls). In their cohort, the lesion-to-background ratio as determined on [^11^C]PIB PET/MR showed 92.3% sensitivity and 100% specificity [146]. Of note, in their paper both MR- and PET-derived parameters were different between patients with and without CA. Conversely, in a single instance the use of [^18^F]Flutemetamol was associated with a very poor diagnostic performance for cardiac amyloidosis. Even though the number of patients with CA are very limited, PET/MRI opens the perspective of more accurate quantification of amyloid load in the heart.

#### Cardiac sarcoidosis

**Fig. 8:**
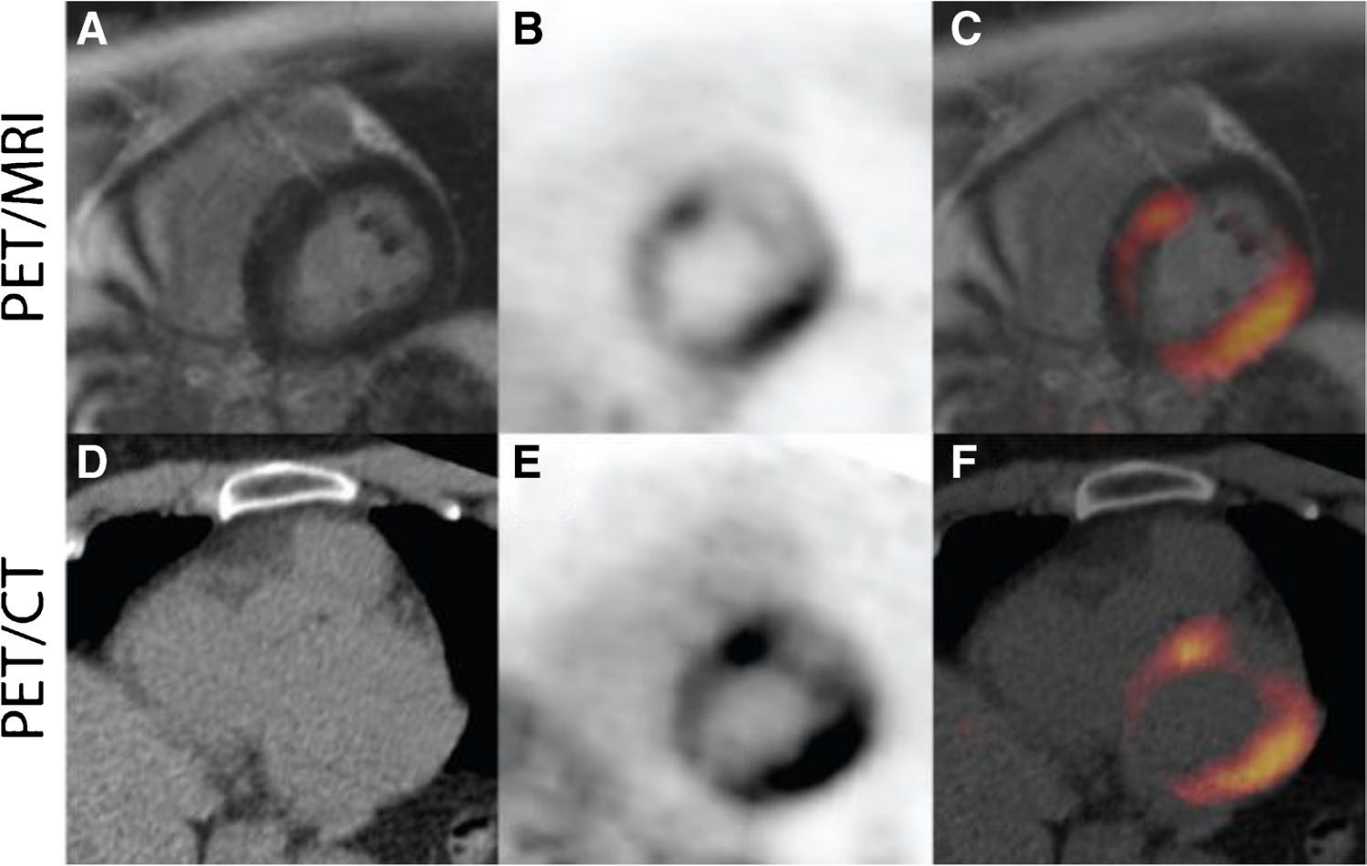
Representative images of hybrid ^18^F-FDG PET/MR (above) and ^18^F-FDG PET/CT (under) in a patient with cardiac sarcoidosis. There is no evidence of signal enhancement on the delayed enhancement MR image (**A**). However, there is a marked increase in FDG uptake, essentially transmural in the lateral wall and anterior septum (**B**, **C**). There is somewhat greater definition as to the extent of this uptake seen on the PET/MR images vs the PET/CT images (**E**, **F**). Reprinted under the terms of the Creative Commons Attribution 4.0 International License (http://creativecommons.org/licenses/by/4.0/) from [147]. No changes were made

Integrated PET/MR evaluation using [^18^F]FDG holds potential in improving accuracy in the diagnosis of active cardiac sarcoidosis compared to stand-alone modalities. The two modalities provide different complementary information: MR LGE informs about areas of myocardial injury which may be active or burnt out, whilst [^18^F]FDG PET informs about ongoing disease activity although it can also reflect false positive physiological uptake in cases where dietary restrictions fail to suppress myocardial glucose utilization. This different information explains why stand-alone PET and MR studies are often affected by inconsistent findings if sequentially performed [147–149] (Fig. 8).

In a recent study, Wicks et al. reported for [^18^F]FDG PET alone sensitivity and specificity of 85% and 56%, respectively (AUC 0.7), while for MR alone sensitivity was 82% and specificity 78% (AUC 0.8). Combining PET and MR, sensitivity increased to 94%, at expenses of a lower specificity (44%), resulting in AUC 0.7. There was poor inter-modality agreement for the location of cardiac abnormalities (k = 0.02). Importantly, PET/MR allowed better prediction of major adverse cardiac events (MACE), with patients demonstrating both PET and MR abnormalities more likely to experience events after 2.2 years follow-up [150].

Another prospective study described 4 patterns of disease that can be identified if hybrid [^18^F]FDG PET/MR is performed: (1) MR + PET + (co-localization of LGE and [^18^F]FDG uptake) provides strong consistent evidence of active cardiac sarcoidosis with ongoing myocardial inflammation and injury; (2) MR-PET- (neither LGE or [^18^F]FDG uptake), provides consistent dual modality evidence that sarcoid is not affecting the heart; (3) MR-PET + (no LGE but [^18^F]FDG uptake), consistent with either false positive myocardial [18F]FDG activity or potentially positive if [^18^F]FDG uptake is localized as can be observed in the early phase; and (4) MR + PET- (LGE with no [^18^F]FDG uptake), consistent with chronic myocardial injury but without ongoing active inflammation [113]. With this approach, hybrid PET/MR provides two important advantages: 1) it yields improved diagnostic accuracy over stand-alone modalities and 2) it provides information on the disease status, which can guide the need for therapy and evaluate subsequent treatment responses [151].

The MR-PET + pattern is often most difficult to interpret. Diffuse [^18^F]FDG uptake in the absence of any LGE is very likely to represent false positive physiological PET uptake, due to failure in suppressing adequately the physiological myocardial [^18^F]FDG uptake [87]. Regional [^18^F]FDG uptake may reflect pathological activity reflecting the higher sensitivity of [^18^F]FDG PET compared to T2-weighted or LGE for imaging the very early stages of the disease, although it can also be observed with physiological false positive [^18^F]FDG uptake if myocardial glucose utilization is only partially suppressed.

### Vascular imaging

High-resolution MRI of the vascular wall relies on the same fundamental principles as other MRI techniques and provides characterisation of the vascular wall on the basis of biophysical and biochemical properties such as chemical composition, water content, physical state, molecular motion, and diffusion. The use of preparatory pulses that specifically null the signal from blood (“black-blood “ sequences) improves contrast between the arterial wall and lumen and helps to delineate the contours of the vascular wall. In addition, multi-contrast MRI consisting of successive MR sequences allows for reasonable differentiation of the different components of atherosclerotic plaques. Furthermore, increased vascularization or inflammation may be identified in the vascular wall on early and delayed T1-weighted images after injection of gadolinium chelates. Complementary to MRI, molecular expression and biological activities inside the vascular wall can be analysed precisely based on the degree of accumulation of various radiopharmaceuticals on PET. The most validated radiopharmaceutical for vascular imaging is [^18^F]FDG. In plaques, the intensity of [^18^F]FDG uptake on PET correlates well with the degree of macrophage infiltration, an important marker of plaque instability [152], but other tracers such as [^18^F]Fluoride targeting microcalcifications [153], [^68^ Ga]DOTA-TATE [154] binding to somatostatin receptors and [^68^ Ga]Pentixafor on chemochin receptor 4 (CXCR4) [155, 156] expressed on activated macrophages have been proposed for the detection of high-risk atherosclerotic plaques.

Combined PET/MRI presents several advantages for vascular imaging. The ability to perform both MRI and PET on the same gantry improves co-registration by avoiding changes in position, in particular of the neck for carotid imaging, that exist when acquisitions are performed on separate PET and MRI scanners. Consequently, the localisation of tracer uptake in the arterial wall is more accurate and can be better delineated from adjacent soft tissue structures. Clinical studies using PET/MRI for the imaging of atherosclerotic plaques are increasing, however, remain rare likely due the limited access to this imaging technology.

The results of whole-body [^18^F]FDG PET/MRI in 755 individuals with subclinical atherosclerosis showed that both plaque number and the number of plaques with high [^18^F]FDG uptake increased with the number of cardiovascular risk factors. Individuals with increased arterial [^18^F]FDG uptake had higher plaque burden (both number and volume) than individuals without arterial inflammation. Patients presenting with embolic stroke of undetermined source, complicated atherosclerotic plaques on MRI were more prevalent in the ipsilateral carotid compared to the contralateral carotid (39 vs 0%), and showed higher [^18^F]FDG uptake than uncomplicated plaque [157]. In view of these promising results, clinical studies specifically investigating the incremental value of combined PET/MR approach over PET/CT are highly warranted.

MR angiography and [^18^F]FDG PET are increasingly used for the noninvasive evaluation of patients with suspected LVV, capitalizing on the ability of MR angiography to depict intramural vessel wall edema and wall thickening, and on the high sensitivity of [^18^F]FDG PET to detect vascular inflammation [158]. In a first paper on a small patients’ sample, [^18^F]FDG PET/MR proved superior to standard inflammatory markers to characterize severe or non-severe LVV [159]. Subsequent evidence confirmed a possible role for [^18^F]FDG PET/MR in the assessment of LVV especially in young patients, owing to reduced radiation exposure compared to standard PET fused with ceCT [160]. Also, a recent study investigated the accuracy of somatostatin receptor PET fused with MR [114], showing that the findings on [^68^ Ga]DOTA-TATE PET/MRI are consistent with those of [^18^F]FDG PET/CT, but with easier visualization of coronary, myocardial, and intracranial artery involvement due to very low background signal in the brain and heart. These first clinical studies suggest at least a non-inferiority of PET/MR compared to PET/CT, which should be confirmed in trials focused on clinical outcome.

### Summary of clinical scenarios. Clinical consensus statement by EANM and EACVI

Different clinical scenarios are summarized in Table 3, along with suggested MR sequences for each indication. Whenever available, hybrid PET/MR should be preferred over stand-alone modalities in the assessment of myocardial viability, cardiac masses, cardiac sarcoidosis, myocarditis and cardiac amyloidosis. Dixon sequences for attenuation correction, cine images and LGE should be included in all MR protocols; stress perfusion and T2-weighted images may be added for the evaluation of myocardial viability and cardiac masses. For cardiac sarcoidosis or myocarditis, T2-weighted images are mandatory, and native T1-mapping and post-contrast T1 mapping for the assessment of ECV may be suggested. Finally, for cardiac amyloidosis, native T1-mapping, LGE and post-contrast T1-mapping are advised.

**Table 3:** Summary of the proposed MR-sequences for combined PET/MR imaging across different clinical indications

Clinical Indication for PET/MR	Dixon sequence for attenuation corection	Cine Images	Perfusion sequence	T2- weighted images	Native T1 mapping	Late Gadolinium Enhancement	Post contrast T1 mapping for ECV
Myocardial Viability	required	required	adequate	adequate	nonappropriate	required	nonappropriate
Cardiac masses	required	required	required	adequate	nonappropriate	required	nonappropriate
Cardiac sarcoidosis/ myocarditis	required	required	nonappropriate	required	adequate	required	adequate
Cardiac amyloidosis	required	required	nonappropriate	nonappropriate	required	required	required

## Perspectives and future agenda

Hybrid imaging has several advantages compared to stand-alone modalities. Besides an improvement in the diagnostic accuracy in diverse cardiovascular diseases, the possibility to simultaneously obtain functional and morphologic information allows investigation of different but complementary aspects of a particular pathologic condition, thereby potentially providing improved, tailored diagnostic and prognostic assessments.

However, hybrid imaging does not represent the “Holy Grail” yet. As a matter of fact, two major issues have to be taken into account when a hybrid approach is considered: higher costs and higher radiation burden if a CT-component is associated.

In view of higher costs for hybrid systems, advances in terms of diagnostic accuracy and/or prognostic assessment should be proven to be cost-effective. To date, there is a lack of studies investigating the economic sustainability of hybrid systems, especially in the case of PET/MR. On the other side, hybrid PET/CT systems are being increasingly embedded in clinical practice, hence suggesting their use in most situations. As increasing evidence in the literature shows advantages for PET/MR systems over stand-alone modalities in specific categories of patients, future studies are now required to determine whether the added benefit of hybrid PET/MRI is worth the associated economic cost. It should be noted that the hybrid PET/MR approach results in significant radiation dose reduction (https://www-pub.iaea.org/MTCD/Publications/PDF/PUB1976_web.pdf), with evident advantages in young patients requiring repeated imaging. Also importantly, department workloads can be optimized and patients can undergo the two modalities in a single instance, without the need for coming again to the radiology department. This latter aspect represents an important improvement in the patient care pathway and patients' quality of life.

Given their high cost, the deployment of PET/MR scanners remains limited. While this aspect limits the wide applicability of our PET/MR recommendations, still in centers wherein such technology is available, clinicians should not refrain from performing hybrid PET/MR imaging. In this context, there is a debate as to whether images should be jointly interpreted by a nuclear medicine physician and a radiologist. A joint interpretation should be at first suggested, capitalizing of the experience and background of specialists in diverse disciplines. This is also consistent with the tendency toward a comprehensive discussion of complex conditions in clinical boards featuring clinical and imaging experts.

Regarding radiation exposure, it is important to select the most appropriate CT protocol and optimize the CT acquisition parameters to limit the radiation exposure of patients **A**s **L**ow **A**s **R**easonably **A**chievable (ALARA). As a matter of fact, the advantage of performing combined imaging should be well balanced with the increase in radiation burden due to possibly less efficient CT technologies on hybrid vs. stand-alone systems. In this context, a trade-off between dose reduction and adequate image quality should be pursued. While unnecessary radiation exposure should be avoided, an additional radiation dose can be accepted if hybrid images are of sufficient quality to yield improved diagnostic accuracy or prognostic value. Of note, the possibility of risk-stratifying patients bears great importance in the choice of the most appropriate therapy, which results in improved outcome.

From this point of view, it can be maintained that combined nuclear and CT acquisitions allow for faster imaging protocols and improved image fusion. This aspect should not be underestimated, as incorrect co-registration between functional and anatomical imaging is prone to error in the visual and semiquantitative assessment of cardiac imaging [24].

## Conclusion

In summary, the use of hybrid PET/CT and PET/MR should be encouraged in selected patients who may benefit from improved diagnostic or prognostic accuracy, by choosing the most appropriate protocol. Furthermore, new applications for e.g. PET/MR may be envisioned, as in the detection of chronic low-grade inflammation disease, wherein stand-alone modalities are not expected to prove useful. By increasing the deployment of hybrid systems, it is also to expect that costs can be reduced, thus enhancing the benefit of hybrid imaging. Finally, the development of new tracers may enhance the need for a hybrid approach. While many clinical applications still need a validation, especially about cost-effectiveness analysis, times are already ripe to use this approach in many clinical conditions, capitalizing on the possibility to see a disease from multiple point of views with the largest amount of information.

## Liability statement

This clinical consensus statement summarizes the views of the EANM Cardiovascular Committee and the specialists for the nuclear-CT section of EACVI. It reflects advices for which the EANM and EACVI cannot be held responsible. The advices should be taken into context of good practice of nuclear medicine and do not substitute for national and international legal or regulatory provisions.

## Data Availability

The data are available upon request at the corresponding author’s address.

## References

[1] Gaemperli O, Saraste A, Knuuti J. Cardiac hybrid imaging. Eur Heart J Cardiovasc Imaging. 2012;13(1):51–60. 10.1093/ejechocard/jer240. 22094239 10.1093/ejechocard/jer240

[2] Guaricci AI, Neglia D, Acampa W, Andreini D, Baggiano A, Bianco F, et al. Computed tomography and nuclear medicine for the assessment of coronary inflammation: clinical applications and perspectives. J Cardiovasc Med (Hagerstown). 2023;24(Suppl 1):e67-76. 10.2459/JCM.0000000000001433. 37052223 10.2459/JCM.0000000000001433

[3] Whittington B, Dweck MR, van Beek EJR, Newby D, Williams MC. PET-MRI of coronary artery disease. J Magn Reson Imaging. 2023;57(5):1301–11. 10.1002/jmri.28554. 36524452 10.1002/jmri.28554

[4] Kwiecinski J, Wolny R, Chwala A, Slomka P. Advances in the assessment of coronary artery disease activity with PET/CT and CTA. Tomography. 2023;9(1):328–41. 10.3390/tomography9010026. 36828378 10.3390/tomography9010026 PMC9962109

[5] Kirienko M, Erba PA, Chiti A, Sollini M. Hybrid PET/MRI in infection and inflammation: an update about the latest available literature evidence. Semin Nucl Med. 2023;53(1):107–24. 10.1053/j.semnuclmed.2022.10.005. 36369091 10.1053/j.semnuclmed.2022.10.005

[6] Kaufmann PA. Cardiac PET/MR: big footprint-small step. J Nucl Cardiol. 2015;22(2):225–6. 10.1007/s12350-015-0089-4. 25711286 10.1007/s12350-015-0089-4

[7] Santana CA, Garcia EV, Faber TL, Sirineni GK, Esteves FP, Sanyal R, et al. Diagnostic performance of fusion of myocardial perfusion imaging (MPI) and computed tomography coronary angiography. J Nucl Cardiol. 2009;16(2):201–11. 10.1007/s12350-008-9019-z. 19156478 10.1007/s12350-008-9019-z PMC3086676

[8] Slomka PJ, Cheng VY, Dey D, Woo J, Ramesh A, Van Kriekinge S, et al. Quantitative analysis of myocardial perfusion SPECT anatomically guided by coregistered 64-slice coronary CT angiography. J Nucl Med. 2009;50(10):1621–30. 10.2967/jnumed.109.063982. 19759104 10.2967/jnumed.109.063982 PMC3530406

[9] Pazhenkottil AP, Nkoulou RN, Ghadri JR, Herzog BA, Buechel RR, Küest SM, et al. Prognostic value of cardiac hybrid imaging integrating single-photon emission computed tomography with coronary computed tomography angiography. Eur Heart J. 2011;32(12):1465–71. 10.1093/eurheartj/ehr047. 21320906 10.1093/eurheartj/ehr047

[10] Kajander S, Joutsiniemi E, Saraste M, Pietilä M, Ukkonen H, Saraste A, et al. Cardiac positron emission tomography/computed tomography imaging accurately detects anatomically and functionally significant coronary artery disease. Circulation. 2010;122(6):603–13. 10.1161/CIRCULATIONAHA.109.915009. 20660808 10.1161/CIRCULATIONAHA.109.915009

[11] Maaniitty T, Stenström I, Bax JJ, Uusitalo V, Ukkonen H, Kajander S, et al. Prognostic value of coronary CT angiography with selective PET perfusion imaging in coronary artery disease. JACC Cardiovasc Imaging. 2017;10(11):1361–70. 10.1016/j.jcmg.2016.10.025. 28528146 10.1016/j.jcmg.2016.10.025

[12] Gitsioudis G, Marwan M, Schneider S, Schmermund A, Korosoglou G, Hausleiter J, et al. A systematic report on non-coronary cardiac CTA in 1097 patients from the German cardiac CT registry. Eur J Radiol. 2020;130:109136. 10.1016/j.ejrad.2020.109136. 32634756 10.1016/j.ejrad.2020.109136

[13] Abbara S, Blanke P, Maroules CD, Cheezum M, Choi AD, Han BK, et al. SCCT guidelines for the performance and acquisition of coronary computed tomographic angiography: a report of the society of cardiovascular computed tomography guidelines committee: endorsed by the North American society for cardiovascular imaging (NASCI). J Cardiovasc Comput Tomogr. 2016;10(6):435–49. 10.1016/j.jcct.2016.10.002. 27780758 10.1016/j.jcct.2016.10.002

[14] Verberne HJ, Acampa W, Anagnostopoulos C, Ballinger J, Bengel F, De Bondt P, et al. EANM procedural guidelines for radionuclide myocardial perfusion imaging with SPECT and SPECT/CT: 2015 revision. Eur J Nucl Med Mol Imaging. 2015;42(12):1929–40. 10.1007/s00259-015-3139-x. 26290421 10.1007/s00259-015-3139-x PMC4589547

[15] Pontone G, Rossi A, Guglielmo M, Dweck MR, Gaemperli O, Nieman K, et al. Clinical applications of cardiac computed tomography: a consensus paper of the European Association of Cardiovascular Imaging-part I. Eur Heart J Cardiovasc Imaging. 2022;23(3):299–314. 10.1093/ehjci/jeab293. 35076061 10.1093/ehjci/jeab293 PMC8863074

[16] Pontone G, Rossi A, Guglielmo M, Dweck MR, Gaemperli O, Nieman K, et al. Clinical applications of cardiac computed tomography: a consensus paper of the European association of cardiovascular imaging-part II. Eur Heart J Cardiovasc Imaging. 2022;23(4):e136–61. 10.1093/ehjci/jeab292. 35175348 10.1093/ehjci/jeab292 PMC8944330

[17] Hausleiter J, Meyer T, Hermann F, Hadamitzky M, Krebs M, Gerber TC, et al. Estimated radiation dose associated with cardiac CT angiography. JAMA. 2009;301(5):500–7. 10.1001/jama.2009.54. 19190314 10.1001/jama.2009.54

[18] Marwan M, Achenbach S, Korosoglou G, Schmermund A, Schneider S, Bruder O, et al. German cardiac CT registry: indications, procedural data and clinical consequences in 7061 patients undergoing cardiac computed tomography. Int J Cardiovasc Imaging. 2018;34(5):807–19. 10.1007/s10554-017-1282-0. 29197025 10.1007/s10554-017-1282-0

[19] Gimelli A, Achenbach S, Buechel RR, Edvardsen T, Francone M, Gaemperli O, et al. Strategies for radiation dose reduction in nuclear cardiology and cardiac computed tomography imaging: a report from the European association of cardiovascular imaging (EACVI), the cardiovascular committee of European association of nuclear medicine (EANM), and the European society of cardiovascular radiology (ESCR). Eur Heart J. 2018;39(4):286–96. 10.1093/eurheartj/ehx582. 29059384 10.1093/eurheartj/ehx582

[20] Voros S, Qian Z. Agatston score tried and true: by contrast, can we quantify calcium on CTA. J Cardiovasc Comput Tomogr. 2012;6(1):45–7. 10.1016/j.jcct.2011.12.002. 22264631 10.1016/j.jcct.2011.12.002

[21] Kędzierski B, Macek P, Dziadkowiec-Macek B, Truszkiewicz K, Poręba R, Gać P. Radiation doses in cardiovascular computed tomography. Life (Basel). 2023;13(4):990. 10.3390/life13040990. 37109519 10.3390/life13040990 PMC10141413

[22] Jang J, Jung SE, Jeong WK, Lim YS, Choi JI, Park MY, et al. Radiation doses of various CT Protocols: a multicenter longitudinal observation study. J Korean Med Sci. 2016;31 Suppl 1(Suppl 1):S24–31. 10.3346/jkms.2016.31.S1.S24. 10.3346/jkms.2016.31.S1.S24 PMC4756338 26908984

[23] Martinez-Möller A, Souvatzoglou M, Navab N, Schwaiger M, Nekolla SG. Artifacts from misaligned CT in cardiac perfusion PET/CT studies: frequency, effects, and potential solutions. J Nucl Med. 2007;48(2):188–93. 17268013

[24] Lautamäki R, Brown TL, Merrill J, Bengel FM. CT-based attenuation correction in (82)Rb-myocardial perfusion PET-CT: incidence of misalignment and effect on regional tracer distribution. Eur J Nucl Med Mol Imaging. 2008;35(2):305–10. 10.1007/s00259-007-0607-y. 17909791 10.1007/s00259-007-0607-y

[25] Goetze S, Brown TL, Lavely WC, Zhang Z, Bengel FM. Attenuation correction in myocardial perfusion SPECT/CT: effects of misregistration and value of reregistration. J Nucl Med. 2007;48(7):1090–5. 10.2967/jnumed.107.040535. 17574985 10.2967/jnumed.107.040535

[26] Gould KL, Pan T, Loghin C, Johnson NP, Guha A, Sdringola S. Frequent diagnostic errors in cardiac PET/CT due to misregistration of CT attenuation and emission PET images: a definitive analysis of causes, consequences, and corrections. J Nucl Med. 2007;48(7):1112–21. 10.2967/jnumed.107.039792. 17574974 10.2967/jnumed.107.039792

[27] Loghin C, Sdringola S, Gould KL. Common artifacts in PET myocardial perfusion images due to attenuation-emission misregistration: clinical significance, causes, and solutions. J Nucl Med. 2004;45(6):1029–39. 15181138

[28] Presotto L. The long fight against motion artifacts in cardiac PET. J Nucl Cardiol. 2022;29(1):69–71. 10.1007/s12350-020-02232-y. 32557239 10.1007/s12350-020-02232-y

[29] Armstrong IS, Hayden C, Memmott MJ, Arumugam P. A preliminary evaluation of a high temporal resolution data-driven motion correction algorithm for rubidium-82 on a SiPM PET-CT system. J Nucl Cardiol. 2022;29(1):56–68. 10.1007/s12350-020-02177-2. 32440990 10.1007/s12350-020-02177-2 PMC8873161

[30] Caobelli F, Akin M, Thackeray JT, Brunkhorst T, Widder J, Berding G, et al. Diagnostic accuracy of cadmium-zinc-telluride-based myocardial perfusion SPECT: impact of attenuation correction using a co-registered external computed tomography. Eur Heart J Cardiovasc Imaging. 2016;17(9):1036–43. 10.1093/ehjci/jev312. 26628617 10.1093/ehjci/jev312

[31] Chopra S, Singh SS, Sood A, Parmar M, Parihar AS, Vadi SK, et al. Comparison of positional artifacts in myocardial perfusion imaging in supine and semi-reclining position using dedicated D-SPECT cardiac camera: validation using CT based attenuation correction. J Nucl Cardiol. 2023. 10.1007/s12350-023-03210-w. 36849635 10.1007/s12350-023-03210-w

[32] Hamill JJ, Brunken RC, Bybel B, DiFilippo FP, Faul DD. A knowledge-based method for reducing attenuation artefacts caused by cardiac appliances in myocardial PET/CT. Phys Med Biol. 2006;51(11):2901–18. 10.1088/0031-9155/51/11/015. 16723774 10.1088/0031-9155/51/11/015

[33] Ghafarian P, Aghamiri SM, Ay MR, Rahmim A, Schindler TH, Ratib O, et al. Is metal artefact reduction mandatory in cardiac PET/CT imaging in the presence of pacemaker and implantable cardioverter defibrillator leads. Eur J Nucl Med Mol Imaging. 2011;38(2):252–62. 10.1007/s00259-010-1635-6. 20959974 10.1007/s00259-010-1635-6

[34] Erba PA, Lancellotti P, Vilacosta I, Gaemperli O, Rouzet F, Hacker M, et al. Recommendations on nuclear and multimodality imaging in IE and CIED infections. Eur J Nucl Med Mol Imaging. 2018;45(10):1795–815. 10.1007/s00259-018-4025-0. 29799067 10.1007/s00259-018-4025-0

[35] Conti D, Baruffaldi F, Erani P, Festa A, Durante S, Santoro M. Dual-energy computed tomography applications to reduce metal artifacts in hip prostheses: a phantom study. Diagnostics (Basel). 2022;13(1):50. 10.3390/diagnostics13010050. 36611342 10.3390/diagnostics13010050 PMC9853491

[36] Johansen A, Grupe P, Veje A, Braad PE, Høilund-Carlsen PF. Scatter and attenuation correction changes interpretation of gated myocardial perfusion imaging. Eur J Nucl Med Mol Imaging. 2004;31(8):1152–9. 10.1007/s00259-004-1481-5. 15118845 10.1007/s00259-004-1481-5

[37] Sharma P, Patel CD, Karunanithi S, Maharjan S, Malhotra A. Comparative accuracy of CT attenuation-corrected and non-attenuation-corrected SPECT myocardial perfusion imaging. Clin Nucl Med. 2012;37(4):332–8. 10.1097/RLU.0b013e31823ea16b. 22391700 10.1097/RLU.0b013e31823ea16b

[38] Greenland P, Blaha MJ, Budoff MJ, Erbel R, Watson KE. Coronary calcium score and cardiovascular risk. J Am Coll Cardiol. 2018;72(4):434–47. 10.1016/j.jacc.2018.05.027. 30025580 10.1016/j.jacc.2018.05.027 PMC6056023

[39] Shaw LJ, Narula J. Risk assessment and predictive value of coronary artery disease testing. J Nucl Med. 2009;50(8):1296–306. 10.2967/jnumed.108.059592. 19652216 10.2967/jnumed.108.059592

[40] Clerc OF, Frey SM, Honegger U, Amrein MLF, Caobelli F, Haaf P, et al. Coronary artery calcium score and pre-test probabilities as gatekeepers to predict and rule out perfusion defects in positron emission tomography. J Nucl Cardiol. 2023. 10.1007/s12350-023-03322-3. 37415007 10.1007/s12350-023-03322-3 PMC10682222

[41] Allio IR, Caobelli F, Popescu CE, Haaf P, Alberts I, Frey SM, et al. Low-dose coronary artery calcium scoring compared to the standard protocol. J Nucl Cardiol. 2023;30(3):1191–8. 10.1007/s12350-022-03120-3. 36289163 10.1007/s12350-022-03120-3 PMC10261226

[42] Juarez-Orozco LE, Martinez-Manzanera O, van der Zant FM, Knol RJJ, Knuuti J. Deep learning in quantitative PET myocardial perfusion imaging: a study on cardiovascular event prediction. JACC Cardiovasc Imaging. 2020;13(1 Pt 1):180–2. 10.1016/j.jcmg.2019.08.009. 31607660 10.1016/j.jcmg.2019.08.009

[43] Mitchell JD, Fergestrom N, Gage BF, Paisley R, Moon P, Novak E, et al. Impact of statins on cardiovascular outcomes following coronary artery calcium scoring. J Am Coll Cardiol. 2018;72(25):3233–42. 10.1016/j.jacc.2018.09.051. 30409567 10.1016/j.jacc.2018.09.051 PMC6309473

[44] Miller RJH, Han D, Singh A, Pieszko K, Slomka PJ, Gransar H, et al. Relationship between ischaemia, coronary artery calcium scores, and major adverse cardiovascular events. Eur Heart J Cardiovasc Imaging. 2022;23(11):1423–33. 10.1093/ehjci/jeac082. 35608211 10.1093/ehjci/jeac082

[45] Aljizeeri A, Ahmed AI, Alfaris MA, Ahmed D, Farea J, Elneama A, et al. Myocardial flow reserve and coronary calcification in prognosis of patients with suspected coronary artery disease. JACC Cardiovasc Imaging. 2021;14(12):2443–52. 10.1016/j.jcmg.2021.01.024. 33744156 10.1016/j.jcmg.2021.01.024

[46] Moser PT, Schernthaner R, Loewe C, Strassl A, Denzinger F, Faby S, et al. Evaluation of perivascular fat attenuation with coronary CT angiography in cardiac transplantation patients: an imaging biomarker candidate for prediction of cardiac mortality and re-transplantation. Eur Radiol. 2023;33(9):6299–307. 10.1007/s00330-023-09614-z. 37072507 10.1007/s00330-023-09614-z PMC10415448

[47] Berman DS, Wong ND, Gransar H, Miranda-Peats R, Dahlbeck J, Hayes SW, et al. Relationship between stress-induced myocardial ischemia and atherosclerosis measured by coronary calcium tomography. J Am Coll Cardiol. 2004;44(4):923–30. 10.1016/j.jacc.2004.06.042. 15312881 10.1016/j.jacc.2004.06.042

[48] Anand DV, Lim E, Raval U, Lipkin D, Lahiri A. Prevalence of silent myocardial ischemia in asymptomatic individuals with subclinical atherosclerosis detected by electron beam tomography. J Nucl Cardiol. 2004;11(4):450–7. 10.1016/j.nuclcard.2004.06.125. 15295414 10.1016/j.nuclcard.2004.06.125

[49] Mc Ardle BA, Dowsley TF, deKemp RA, Wells GA, Beanlands RS. Does rubidium-82 PET have superior accuracy to SPECT perfusion imaging for the diagnosis of obstructive coronary disease?: A systematic review and meta-analysis. J Am Coll Cardiol. 2012;60(18):1828–37. 10.1016/j.jacc.2012.07.038. 23040573 10.1016/j.jacc.2012.07.038

[50] Jaarsma C, Leiner T, Bekkers SC, Crijns HJ, Wildberger JE, Nagel E, et al. Diagnostic performance of noninvasive myocardial perfusion imaging using single-photon emission computed tomography, cardiac magnetic resonance, and positron emission tomography imaging for the detection of obstructive coronary artery disease: a meta-analysis. J Am Coll Cardiol. 2012;59(19):1719–28. 10.1016/j.jacc.2011.12.040. 22554604 10.1016/j.jacc.2011.12.040

[51] Cury RC, Leipsic J, Abbara S, Achenbach S, Berman D, Bittencourt M, et al. CAD-RADS™ 2.0 - 2022 Coronary Artery Disease - Reporting and Data System.: An expert consensus document of the Society of Cardiovascular Computed Tomography (SCCT), the American College of Cardiology (ACC), the American College of Radiology (ACR) and the North America Society of Cardiovascular Imaging (NASCI). J Am Coll Radiol. 2022;19(11):1185–212. 10.1016/j.jacr.2022.09.012. 36436841 10.1016/j.jacr.2022.09.012

[52] Maaniitty T, Jaakkola S, Saraste A, Knuuti J. Hybrid coronary computed tomography angiography and positron emission tomography myocardial perfusion imaging in evaluation of recurrent symptoms after coronary artery bypass grafting. Eur Heart J Cardiovasc Imaging. 2019;20(11):1298–304. 10.1093/ehjci/jey160. 30388202 10.1093/ehjci/jey160

[53] Jones DA, Beirne AM, Kelham M, Rathod KS, Andiapen M, Wynne L, et al. Computed tomography cardiac angiography before invasive coronary angiography in patients with previous bypass surgery: the BYPASS-CTCA trial. Circulation. 2023;148(18):1371–80. 10.1161/CIRCULATIONAHA.123.064465. 37772419 10.1161/CIRCULATIONAHA.123.064465 PMC11139242

[54] Cerqueira MD, Weissman NJ, Dilsizian V, Jacobs AK, Kaul S, Laskey WK, et al. Standardized myocardial segmentation and nomenclature for tomographic imaging of the heart. A statement for healthcare professionals from the cardiac imaging committee of the council on clinical cardiology of the American heart association. Circulation. 2002;105(4):539–42. 10.1161/hc0402.102975. 11815441 10.1161/hc0402.102975

[55] Javadi MS, Lautamäki R, Merrill J, Voicu C, Epley W, McBride G, et al. Definition of vascular territories on myocardial perfusion images by integration with true coronary anatomy: a hybrid PET/CT analysis. J Nucl Med. 2010;51(2):198–203. 10.2967/jnumed.109.067488. 20080895 10.2967/jnumed.109.067488

[56] Bom MJ, Schumacher SP, Driessen RS, Raijmakers PG, Everaars H, van Diemen PA, et al. Impact of individualized segmentation on diagnostic performance of quantitative positron emission tomography for haemodynamically significant coronary artery disease. Eur Heart J Cardiovasc Imaging. 2019;20(5):525–32. 10.1093/ehjci/jey201. 30590493 10.1093/ehjci/jey201

[57] Caobelli F. Left ventricular segmentation in myocardial perfusion positron emission tomography: tailor-made or prêt-à-porter. Eur Heart J Cardiovasc Imaging. 2019;20(5):502–3. 10.1093/ehjci/jey216. 30608576 10.1093/ehjci/jey216

[58] AlBadri A, Piccinelli M, Cho SG, Lee JM, Jaber W, De Cecco CN, et al. Rationale and design of the quantification of myocardial blood flow using dynamic PET/CTA-fused imagery (DEMYSTIFY) to determine physiological significance of specific coronary lesions. J Nucl Cardiol. 2020;27(3):1030–9. 10.1007/s12350-020-02052-0. 32026327 10.1007/s12350-020-02052-0 PMC7332386

[59] Namdar M, Hany TF, Koepfli P, Siegrist PT, Burger C, Wyss CA, et al. Integrated PET/CT for the assessment of coronary artery disease: a feasibility study. J Nucl Med. 2005;46(6):930–5. 15937302

[60] Danad I, Raijmakers PG, Knaapen P. Diagnosing coronary artery disease with hybrid PET/CT: it takes two to tango. J Nucl Cardiol. 2013;20(5):874–90. 10.1007/s12350-013-9753-8. 23842709 10.1007/s12350-013-9753-8

[61] Groves PH, Pomfrett C, Marlow M. Review of the role of NICE in promoting the adoption of innovative cardiac technologies. Heart. 2018;104(22):1817–22. 10.1136/heartjnl-2018-313256. 29773657 10.1136/heartjnl-2018-313256 PMC6241610

[62] Thomassen A, Petersen H, Diederichsen AC, Mickley H, Jensen LO, Johansen A, et al. Hybrid CT angiography and quantitative 15O-water PET for assessment of coronary artery disease: comparison with quantitative coronary angiography. Eur J Nucl Med Mol Imaging. 2013;40(12):1894–904. 10.1007/s00259-013-2519-3. 23982453 10.1007/s00259-013-2519-3

[63] Rizvi A, Han D, Danad I, Ó Hartaigh B, Lee JH, Gransar H, et al. Diagnostic performance of hybrid cardiac imaging methods for assessment of obstructive coronary artery disease compared with stand-alone coronary computed tomography angiography: a meta-analysis. JACC Cardiovasc Imaging. 2018;11(4):589–99. 10.1016/j.jcmg.2017.05.020. 10.1016/j.jcmg.2017.05.020 PMC5808913 28823745

[64] Danad I, Raijmakers PG, Driessen RS, Leipsic J, Raju R, Naoum C, et al. Comparison of coronary CT angiography, SPECT, PET, and hybrid imaging for diagnosis of ischemic heart disease determined by fractional flow reserve. JAMA Cardiol. 2017;2(10):1100–7. 10.1001/jamacardio.2017.2471. 28813561 10.1001/jamacardio.2017.2471 PMC5710451

[65] Westra J, Rasmussen LD, Eftekhari A, Winther S, Karim SR, Johansen JK, et al. Coronary artery stenosis evaluation by angiography-derived FFR: validation by positron emission tomography and invasive thermodilution. JACC Cardiovasc Imaging. 2023S1936–878X(23)00105. 10.1016/j.jcmg.2023.02.008. 10.1016/j.jcmg.2023.02.008 37052562

[66] Driessen RS, Bom MJ, van Diemen PA, Schumacher SP, Leonora RM, Everaars H, et al. Incremental prognostic value of hybrid [15O]H2O positron emission tomography-computed tomography: combining myocardial blood flow, coronary stenosis severity, and high-risk plaque morphology. Eur Heart J Cardiovasc Imaging. 2020;21(10):1105–13. 10.1093/ehjci/jeaa192. 32959061 10.1093/ehjci/jeaa192 PMC7971168

[67] Sciagrà R, Lubberink M, Hyafil F, Saraste A, Slart RHJA, Agostini D, et al. EANM procedural guidelines for PET/CT quantitative myocardial perfusion imaging. Eur J Nucl Med Mol Imaging. 2021;48(4):1040–69. 10.1007/s00259-020-05046-9. 33135093 10.1007/s00259-020-05046-9 PMC7603916

[68] Carsuzaa T, Thibault F, Bailly M. Gated tomographic radionuclide angiography using 3D-Ring CZT StarGuide SPECT/CT head-to-head comparison with a cardiac-dedicated CZT camera: first clinical use and validation. Clin Nucl Med. 2022;47(7):e515–7. 10.1097/RLU.0000000000004153. 35353756 10.1097/RLU.0000000000004153

[69] Ben Bouallègue F, Maïmoun L, Kucharczak F, Le Fur P, Vauchot F, Hay B, et al. Left ventricle function assessment using gated first-pass ^18^F-FDG PET: validation against equilibrium radionuclide angiography. J Nucl Cardiol. 2021;28(2):594–603. 10.1007/s12350-019-01731-x. 31044403 10.1007/s12350-019-01731-x

[70] Zaidi H, Hasegawa B. Determination of the attenuation map in emission tomography. J Nucl Med. 2003;44(2):291–315. 12571222

[71] Slomka PJ, Diaz-Zamudio M, Dey D, Motwani M, Brodov Y, Choi D, et al. Automatic registration of misaligned CT attenuation correction maps in Rb-82 PET/CT improves detection of angiographically significant coronary artery disease. J Nucl Cardiol. 2015;22(6):1285–95. 10.1007/s12350-014-0060-9. 25698471 10.1007/s12350-014-0060-9

[72] Kennedy JA, Israel O, Frenkel A. Directions and magnitudes of misregistration of CT attenuation-corrected myocardial perfusion studies: incidence, impact on image quality, and guidance for reregistration. J Nucl Med. 2009;50(9):1471–8. 10.2967/jnumed.109.062141. 19690038 10.2967/jnumed.109.062141

[73] O’Connor MK, Kemp BJ. Single-photon emission computed tomography/computed tomography: basic instrumentation and innovations. Semin Nucl Med. 2006;36(4):258–66. 10.1053/j.semnuclmed.2006.05.005. 16950143 10.1053/j.semnuclmed.2006.05.005

[74] Delgado V, Ajmone Marsan N, de Waha S, Bonaros N, Brida M, Burri H, et al. 2023 ESC Guidelines for the management of endocarditis. Eur Heart J. 2023ehad193. 10.1093/eurheartj/ehad193. 10.1093/eurheartj/ehad193 37622656

[75] Bourque JM, Birgersdotter-Green U, Bravo PE, Budde RPJ, Chen W, Chu VH, et al. 18F-FDG PET/CT and radiolabeled leukocyte SPECT/CT imaging for the evaluation of cardiovascular infection in the multimodality context. JACC: Cardiovasc Imaging. 2024. 10.1016/j.jcmg.2024.01.004. 10.1016/j.jcmg.2024.01.004 38466252

[76] Bourque JM, Birgersdotter-Green U, Bravo PE, Budde RPJ, Chen W, Chu VH, et al. ^18^F-FDG PET/CT and radiolabeled leukocyte SPECT/CT imaging for the evaluation of cardiovascular infection in the multimodality context: ASNC imaging indications (ASNC I^2^) series expert consensus recommendations from ASNC, AATS, ACC, AHA, ASE, EANM, HRS, IDSA, SCCT, SNMMI, and STS. J Nucl Cardiol. 2024;34:101786. 10.1016/j.nuclcard.2023.101786. 38472038 10.1016/j.nuclcard.2023.101786

[77] Blomström-Lundqvist C, Traykov V, Erba PA, Burri H, Nielsen JC, Bongiorni MG, et al. European heart rhythm association (EHRA) international consensus document on how to prevent, diagnose, and treat cardiac implantable electronic device infections-endorsed by the Heart Rhythm Society (HRS), the Asia Pacific Heart Rhythm Society (APHRS), the Latin American Heart Rhythm Society (LAHRS), International Society for Cardiovascular Infectious Diseases (ISCVID) and the European Society of Clinical Microbiology and Infectious Diseases (ESCMID) in collaboration with the European Association for Cardio-Thoracic Surgery (EACTS). Europace. 2020;22(4):515–49. 10.1093/europace/euz246. 31702000 10.1093/europace/euz246 PMC7132545

[78] Habib G, Lancellotti P, Antunes MJ, Bongiorni MG, Casalta JP, Del Zotti F, et al. 2015 ESC Guidelines for the management of infective endocarditis: the task force for the management of infective endocarditis of the european society of cardiology (ESC). Endorsed by: European association for cardio-thoracic surgery (EACTS), the European association of nuclear medicine (EANM). Eur Heart J. 2015;36(44):3075–128. 10.1093/eurheartj/ehv319. 26320109 10.1093/eurheartj/ehv319

[79] Pizzi MN, Roque A, Cuéllar-Calabria H, Fernández-Hidalgo N, Ferreira-González I, González-Alujas MT, et al. ^18^F-FDG-PET/CTA of prosthetic cardiac valves and valve-tube grafts: infective versus inflammatory patterns. JACC Cardiovasc Imaging. 2016;9(10):1224–7. 10.1016/j.jcmg.2016.05.013. 27639767 10.1016/j.jcmg.2016.05.013

[80] Pizzi MN, Roque A, Fernández-Hidalgo N, Cuéllar-Calabria H, Ferreira-González I, Gonzàlez-Alujas MT, et al. Improving the diagnosis of infective endocarditis in prosthetic valves and intracardiac devices With 18F-Fluordeoxyglucose positron emission tomography/computed tomography angiography: initial results at an infective endocarditis referral center. Circulation. 2015;132(12):1113–26. 10.1161/CIRCULATIONAHA.115.015316. 26276890 10.1161/CIRCULATIONAHA.115.015316

[81] Salaun E, Sportouch L, Barral PA, Hubert S, Lavoute C, Casalta AC, et al. Diagnosis of infective endocarditis after TAVR: value of a multimodality imaging approach. JACC Cardiovasc Imaging. 2018;11(1):143–6. 10.1016/j.jcmg.2017.05.016. 28823740 10.1016/j.jcmg.2017.05.016

[82] Wahadat AR, Tanis W, Swart LE, Scholtens A, Krestin GP, van Mieghem NMDA, et al. Added value of ^18^F-FDG-PET/CT and cardiac CTA in suspected transcatheter aortic valve endocarditis. J Nucl Cardiol. 2021;28(5):2072–82. 10.1007/s12350-019-01963-x. 31792918 10.1007/s12350-019-01963-x PMC8648682

[83] Olivella A, Pizzi MN, Dos-Subirà L, Aguadé-Bruix S, Fernández-Hidalgo N, Cuéllar-Calabria H, et al. Right-sided endocarditis on Contegra tube in a complex cianotic congenital heart disease. J Nucl Cardiol. 2020;27(4):1402–4. 10.1007/s12350-019-01708-w. 30972721 10.1007/s12350-019-01708-w

[84] Signore A, Jamar F, Israel O, Buscombe J, Martin-Comin J, Lazzeri E. Clinical indications, image acquisition and data interpretation for white blood cells and anti-granulocyte monoclonal antibody scintigraphy: an EANM procedural guideline. Eur J Nucl Med Mol Imaging. 2018;45(10):1816–31. 10.1007/s00259-018-4052-x. 29850929 10.1007/s00259-018-4052-x PMC6097781

[85] Rouzet F, Chequer R, Benali K, Lepage L, Ghodbane W, Duval X, et al. Respective performance of 18F-FDG PET and radiolabeled leukocyte scintigraphy for the diagnosis of prosthetic valve endocarditis. J Nucl Med. 2014;55(12):1980–5. 10.2967/jnumed.114.141895. 25453046 10.2967/jnumed.114.141895

[86] Caobelli F, Wollenweber T, Bavendiek U, Kühn C, Schütze C, Geworski L, et al. Simultaneous dual-isotope solid-state detector SPECT for improved tracking of white blood cells in suspected endocarditis. Eur Heart J. 2017;38(6):436–43. 10.1093/eurheartj/ehw231. 27469371 10.1093/eurheartj/ehw231

[87] Genovesi D, Bauckneht M, Altini C, Popescu CE, Ferro P, Monaco L, et al. The role of positron emission tomography in the assessment of cardiac sarcoidosis. Br J Radiol. 2019;92(1100):20190247. 10.1259/bjr.20190247. 31166768 10.1259/bjr.20190247 PMC6724628

[88] Jennette JC, Falk RJ, Bacon PA, Basu N, Cid MC, Ferrario F, et al. 2012 revised international Chapel Hill consensus conference nomenclature of vasculitides. Arthritis Rheum. 2013;65(1):1–11. 10.1002/art.37715. 23045170 10.1002/art.37715

[89] Pugh D, Karabayas M, Basu N, Cid MC, Goel R, Goodyear CS, et al. Large-vessel vasculitis. Nat Rev Dis Primers. 2022;7(1):93. 10.1038/s41572-021-00327-5. 34992251 10.1038/s41572-021-00327-5 PMC9115766

[90] Yamada I, Nakagawa T, Himeno Y, Numano F, Shibuya H. Takayasu arteritis: evaluation of the thoracic aorta with CT angiography. Radiology. 1998;209(1):103–9. 10.1148/radiology.209.1.9769819. 9769819 10.1148/radiology.209.1.9769819

[91] Hur JH, Chun EJ, Kwag HJ, Yoo JY, Kim HY, Kim JJ, et al. CT features of vasculitides based on the 2012 international Chapel Hill consensus conference revised classification. Korean J Radiol. 2017;18(5):786–98. 10.3348/kjr.2017.18.5.786. 28860896 10.3348/kjr.2017.18.5.786 PMC5552462

[92] García-Martínez A, Arguis P, Prieto-González S, Espígol-Frigolé G, Alba MA, Butjosa M, et al. Prospective long term follow-up of a cohort of patients with giant cell arteritis screened for aortic structural damage (aneurysm or dilatation). Ann Rheum Dis. 2014;73(10):1826–32. 10.1136/annrheumdis-2013-203322. 23873881 10.1136/annrheumdis-2013-203322

[93] Koch V, Abt J, Gruenewald LD, Eichler K, D’Angelo T, Martin SS, et al. Systematic evaluation of imaging techniques and baseline characteristics in patients with suspected vasculitis. Eur J Radiol Open. 2022;9:100445. 10.1016/j.ejro.2022.100445. 36262692 10.1016/j.ejro.2022.100445 PMC9574707

[94] Jamar F, Buscombe J, Chiti A, Christian PE, Delbeke D, Donohoe KJ, et al. EANM/SNMMI guideline for 18F-FDG use in inflammation and infection. J Nucl Med. 2013;54(4):647–58. 10.2967/jnumed.112.112524. 23359660 10.2967/jnumed.112.112524

[95] Slart RHJA, Writing G, Reviewer G, Members OEANMC, Members OEANMII, Members of Committees SNMMIC, et al. FDG-PET/CT(A) imaging in large vessel vasculitis and polymyalgia rheumatica: joint procedural recommendation of the EANM, SNMMI, and the PET Interest Group (PIG), and endorsed by the ASNC. Eur J Nucl Med Mol Imaging. 2018;45(7):1250–69. 10.1007/s00259-018-3973-8. 10.1007/s00259-018-3973-8 PMC5954002 29637252

[96] Lariviere D, Benali K, Coustet B, Pasi N, Hyafil F, Klein I, et al. Positron emission tomography and computed tomography angiography for the diagnosis of giant cell arteritis: A real-life prospective study. Medicine (Baltimore). 2016;95(30):e4146. 10.1097/MD.0000000000004146. 27472684 10.1097/MD.0000000000004146 PMC5265821

[97] Lensen KD, Comans EF, Voskuyl AE, van der Laken CJ, Brouwer E, Zwijnenburg AT, et al. Large-vessel vasculitis: interobserver agreement and diagnostic accuracy of 18F-FDG-PET/CT. Biomed Res Int. 2015;2015:914692. 10.1155/2015/914692. 25695092 10.1155/2015/914692 PMC4324480

[98] Bucerius J, Hyafil F, Verberne HJ, Slart RH, Lindner O, Sciagra R, et al. Position paper of the cardiovascular committee of the European association of nuclear medicine (EANM) on PET imaging of atherosclerosis. Eur J Nucl Med Mol Imaging. 2016;43(4):780–92. 10.1007/s00259-015-3259-3. 26678270 10.1007/s00259-015-3259-3 PMC4764627

[99] Nielsen BD, Gormsen LC, Hansen IT, Keller KK, Therkildsen P, Hauge EM. Three days of high-dose glucocorticoid treatment attenuates large-vessel 18F-FDG uptake in large-vessel giant cell arteritis but with a limited impact on diagnostic accuracy. Eur J Nucl Med Mol Imaging. 2018;45(7):1119–28. 10.1007/s00259-018-4021-4. 29671039 10.1007/s00259-018-4021-4

[100] Drent M, Crouser ED, Grunewald J. Challenges of sarcoidosis and its management. N Engl J Med. 2021;385(11):1018–32. 10.1056/NEJMra2101555. 34496176 10.1056/NEJMra2101555

[101] Statement on sarcoidosis. Joint statement of the American thoracic society (ATS), the European respiratory society (ERS) and the World association of sarcoidosis and other granulomatous disorders (WASOG) adopted by the ATS board of directors and by the ers executive committee, february 1999. Am J Respir Crit Care Med. 1999;160(2):736–55. 10.1164/ajrccm.160.2.ats4-99. 10430755 10.1164/ajrccm.160.2.ats4-99

[102] Régis C, Benali K, Rouzet F. FDG PET/CT imaging of sarcoidosis. Semin Nucl Med. 2023;53(2):258–72. 10.1053/j.semnuclmed.2022.08.004. 36870707 10.1053/j.semnuclmed.2022.08.004

[103] Lobert P, Brown RK, Dvorak RA, Corbett JR, Kazerooni EA, Wong KK. Spectrum of physiological and pathological cardiac and pericardial uptake of FDG in oncology PET-CT. Clin Radiol. 2013;68(1):e59-71. 10.1016/j.crad.2012.09.007. 23177651 10.1016/j.crad.2012.09.007

[104] Ozutemiz C, Koksel Y, Froelich JW, Rubin N, Bhargava M, Roukuz H, et al. Comparison of the effect of three different dietary modifications on myocardial suppression in ^18^f-fdg pet/ct evaluation of patients for suspected cardiac sarcoidosis. J Nucl Med. 2021;62(12):1759–67. 10.2967/jnumed.121.261981. 33771904 10.2967/jnumed.121.261981 PMC8612186

[105] Lucinian YA, Martineau P, Poenaru R, Tremblay-Gravel M, Cadrin-Tourigny J, Harel F, et al. FDG-PET/CT and rest myocardial perfusion imaging to predict high-degree atrioventricular block recovery in cardiac sarcoidosis. J Nucl Cardiol. 2023;30(6):2490–500. 10.1007/s12350-023-03306-3. 37258950 10.1007/s12350-023-03306-3

[106] Slart RHJA, Glaudemans AWJM, Gheysens O, Lubberink M, Kero T, Dweck MR, et al. Procedural recommendations of cardiac PET/CT imaging: standardization in inflammatory-, infective-, infiltrative-, and innervation (4Is)-related cardiovascular diseases: a joint collaboration of the EACVI and the EANM. Eur J Nucl Med Mol Imaging. 2021;48(4):1016–39. 10.1007/s00259-020-05066-5. 33106926 10.1007/s00259-020-05066-5 PMC8041672

[107] Perugini E, Guidalotti PL, Salvi F, Cooke RM, Pettinato C, Riva L, et al. Noninvasive etiologic diagnosis of cardiac amyloidosis using 99mTc-3,3-diphosphono-1,2-propanodicarboxylic acid scintigraphy. J Am Coll Cardiol. 2005;46(6):1076–84. 10.1016/j.jacc.2005.05.073. 16168294 10.1016/j.jacc.2005.05.073

[108] Garcia-Pavia P, Rapezzi C, Adler Y, Arad M, Basso C, Brucato A, et al. Diagnosis and treatment of cardiac amyloidosis: a position statement of the ESC working group on myocardial and pericardial diseases. Eur Heart J. 2021;42(16):1554–68. 10.1093/eurheartj/ehab072. 33825853 10.1093/eurheartj/ehab072 PMC8060056

[109] Hanna M, Ruberg FL, Maurer MS, Dispenzieri A, Dorbala S, Falk RH, et al. Cardiac scintigraphy with technetium-99m-labeled bone-seeking tracers for suspected amyloidosis: JACC review topic of the week. J Am Coll Cardiol. 2020;75(22):2851–62. 10.1016/j.jacc.2020.04.022. 32498813 10.1016/j.jacc.2020.04.022

[110] Caobelli F, Braun M, Haaf P, Wild D, Zellweger MJ. Quantitative ^99m^Tc-DPD SPECT/CT in patients with suspected ATTR cardiac amyloidosis: feasibility and correlation with visual scores. J Nucl Cardiol. 2020;27(5):1456–63. 10.1007/s12350-019-01893-8. 31538322 10.1007/s12350-019-01893-8

[111] Prakken NHJ, Besson FL, Borra RJH, Büther F, Buechel RR, Catana C, et al. PET/MRI in practice: a clinical centre survey endorsed by the European Association of Nuclear Medicine (EANM) and the EANM Forschungs GmbH (EARL). Eur J Nucl Med Mol Imaging. 2023;50(10):2927–34. 10.1007/s00259-023-06308-y. 37378857 10.1007/s00259-023-06308-y

[112] Trivieri MG, Dweck MR, Abgral R, Robson PM, Karakatsanis NA, Lala A, et al. ^18^F-Sodium fluoride PET/MR for the assessment of cardiac amyloidosis. J Am Coll Cardiol. 2016;68(24):2712–4. 10.1016/j.jacc.2016.09.953. 27978955 10.1016/j.jacc.2016.09.953 PMC5438164

[113] Dweck MR, Abgral R, Trivieri MG, Robson PM, Karakatsanis N, Mani V, et al. Hybrid magnetic resonance imaging and positron emission tomography with fluorodeoxyglucose to diagnose active cardiac sarcoidosis. JACC Cardiovasc Imaging. 2018;11(1):94–107. 10.1016/j.jcmg.2017.02.021. 28624396 10.1016/j.jcmg.2017.02.021 PMC5995315

[114] Ćorović A, Wall C, Nus M, Gopalan D, Huang Y, Imaz M, et al. Somatostatin receptor PET/MR imaging of inflammation in patients with large vessel vasculitis and atherosclerosis. J Am Coll Cardiol. 2023;81(4):336–54. 10.1016/j.jacc.2022.10.034. 36697134 10.1016/j.jacc.2022.10.034 PMC9883634

[115] Abgral R, Dweck MR, Trivieri MG, Robson PM, Karakatsanis N, Mani V, et al. Clinical utility of combined FDG-PET/MR to assess myocardial disease. JACC Cardiovasc Imaging. 2017;10(5):594–7. 10.1016/j.jcmg.2016.02.029. 27372018 10.1016/j.jcmg.2016.02.029 PMC5199624

[116] Robson PM, Dweck MR, Trivieri MG, Abgral R, Karakatsanis NA, Contreras J, et al. Coronary Artery PET/MR Imaging: Feasibility, Limitations, and Solutions. JACC Cardiovasc Imaging. 2017;10(10 Pt A):1103–12. 10.1016/j.jcmg.2016.09.029. 10.1016/j.jcmg.2016.09.029 PMC5509532 28109921

[117] von Felten E, Benetos G, Patriki D, Benz DC, Rampidis GP, Giannopoulos AA, et al. Myocardial creep-induced misalignment artifacts in PET/MR myocardial perfusion imaging. Eur J Nucl Med Mol Imaging. 2021;48(2):406–13. 10.1007/s00259-020-04956-y. 32681446 10.1007/s00259-020-04956-y PMC7835156

[118] Zhang Z, Chen X, Wan Q, Wang H, Qi N, You Z, et al. A two-stage cardiac PET and late gadolinium enhancement MRI co-registration method for improved assessment of non-ischemic cardiomyopathies using integrated PET/MR. Eur J Nucl Med Mol Imaging. 2022;49(7):2199–208. 10.1007/s00259-022-05681-4. 35031812 10.1007/s00259-022-05681-4

[119] Bogdanovic B, Gafita A, Schachoff S, Eiber M, Cabello J, Weber WA, et al. Almost 10 years of PET/MR attenuation correction: the effect on lesion quantification with PSMA: clinical evaluation on 200 prostate cancer patients. Eur J Nucl Med Mol Imaging. 2021;48(2):543–53. 10.1007/s00259-020-04957-x. 32725538 10.1007/s00259-020-04957-x PMC7835314

[120] Ghosh N, Rimoldi OE, Beanlands RS, Camici PG. Assessment of myocardial ischaemia and viability: role of positron emission tomography. Eur Heart J. 2010;31(24):2984–95. 10.1093/eurheartj/ehq361. 20965888 10.1093/eurheartj/ehq361

[121] Beanlands RS, Hendry PJ, Masters RG, deKemp RA, Woodend K, Ruddy TD. Delay in revascularization is associated with increased mortality rate in patients with severe left ventricular dysfunction and viable myocardium on fluorine 18-fluorodeoxyglucose positron emission tomography imaging. Circulation. 1998;98(19 Suppl):II51–6. 9852880

[122] Nensa F, Poeppel TD, Beiderwellen K, Schelhorn J, Mahabadi AA, Erbel R, et al. Hybrid PET/MR imaging of the heart: feasibility and initial results. Radiology. 2013;268(2):366–73. 10.1148/radiol.13130231. 23651530 10.1148/radiol.13130231

[123] Rischpler C, Langwieser N, Souvatzoglou M, Batrice A, van Marwick S, Snajberk J, et al. PET/MRI early after myocardial infarction: evaluation of viability with late gadolinium enhancement transmurality vs. 18F-FDG uptake. Eur Heart J Cardiovasc Imaging. 2015;16(6):661–9. 10.1093/ehjci/jeu317. 10.1093/ehjci/jeu317 25680385

[124] Vitadello T, Kunze KP, Nekolla SG, Langwieser N, Bradaric C, Weis F, et al. Hybrid PET/MR imaging for the prediction of left ventricular recovery after percutaneous revascularisation of coronary chronic total occlusions. Eur J Nucl Med Mol Imaging. 2020;47(13):3074–83. 10.1007/s00259-020-04877-w. 32472438 10.1007/s00259-020-04877-w PMC7680332

[125] Peretto G, Busnardo E, Ferro P, Palmisano A, Vignale D, Esposito A, et al. Clinical applications of FDG-PET scan in arrhythmic myocarditis. JACC Cardiovasc Imaging. 2022;15(10):1771–80. 10.1016/j.jcmg.2022.02.029. 36202457 10.1016/j.jcmg.2022.02.029

[126] Caobelli F, Cabrero JB, Galea N, Haaf P, Loewe C, Luetkens JA, et al. Cardiovascular magnetic resonance (CMR) and positron emission tomography (PET) imaging in the diagnosis and follow-up of patients with acute myocarditis and chronic inflammatory cardiomyopathy : A review paper with practical recommendations on behalf of the European Society of Cardiovascular Radiology (ESCR). Int J Cardiovasc Imaging. 2023;39(11):2221–35. 10.1007/s10554-023-02927-6. 37682416 10.1007/s10554-023-02927-6 PMC10674005

[127] Nensa F, Poeppel TD, Krings P, Schlosser T. Multiparametric assessment of myocarditis using simultaneous positron emission tomography/magnetic resonance imaging. Eur Heart J. 2014;35(32):2173. 10.1093/eurheartj/ehu086. 24578391 10.1093/eurheartj/ehu086

[128] von Olshausen G, Hyafil F, Langwieser N, Laugwitz KL, Schwaiger M, Ibrahim T. Detection of acute inflammatory myocarditis in Epstein Barr virus infection using hybrid 18F-fluoro-deoxyglucose-positron emission tomography/magnetic resonance imaging. Circulation. 2014;130(11):925–6. 10.1161/CIRCULATIONAHA.114.011000. 25199667 10.1161/CIRCULATIONAHA.114.011000

[129] Piriou N, Sassier J, Pallardy A, Serfaty JM, Trochu JN. Utility of cardiac FDG-PET imaging coupled to magnetic resonance for the management of an acute myocarditis with non-informative endomyocardial biopsy. Eur Heart J Cardiovasc Imaging. 2015;16(5):574. 10.1093/ehjci/jeu319. 25588796 10.1093/ehjci/jeu319

[130] Nensa F, Kloth J, Tezgah E, Poeppel TD, Heusch P, Goebel J, et al. Feasibility of FDG-PET in myocarditis: comparison to CMR using integrated PET/MRI. J Nucl Cardiol. 2018;25(3):785–94. 10.1007/s12350-016-0616-y. 27638745 10.1007/s12350-016-0616-y

[131] Amano J, Nakayama J, Yoshimura Y, Ikeda U. Clinical classification of cardiovascular tumors and tumor-like lesions, and its incidences. Gen Thorac Cardiovasc Surg. 2013;61(8):435–47. 10.1007/s11748-013-0214-8. 23460447 10.1007/s11748-013-0214-8 PMC3732772

[132] Qin C, Shao F, Hu F, Song W, Song Y, Guo J, et al. ^18^F-FDG PET/CT in diagnostic and prognostic evaluation of patients with cardiac masses: a retrospective study. Eur J Nucl Med Mol Imaging. 2020;47(5):1083–93. 10.1007/s00259-019-04632-w. 31807883 10.1007/s00259-019-04632-w

[133] Palaskas N, Thompson K, Gladish G, Agha AM, Hassan S, Iliescu C, et al. Evaluation and management of cardiac tumors. Curr Treat Options Cardiovasc Med. 2018;20(4):29. 10.1007/s11936-018-0625-z. 29556752 10.1007/s11936-018-0625-z

[134] Hendel RC, Patel MR, Kramer CM, Poon M, Hendel RC, Carr JC, et al. ACCF/ACR/SCCT/SCMR/ASNC/NASCI/SCAI/SIR 2006 appropriateness criteria for cardiac computed tomography and cardiac magnetic resonance imaging: a report of the American college of cardiology foundation quality strategic directions committee appropriateness criteria working group, American college of radiology, Society of cardiovascular computed tomography, Society for cardiovascular magnetic resonance, American society of nuclear cardiology, North American society for cardiac imaging, Society for cardiovascular angiography and interventions, and Society of interventional radiology. J Am Coll Cardiol. 2006;48(7):1475–97. 10.1016/j.jacc.2006.07.003. 10.1016/j.jacc.2006.07.003 17010819

[135] Parwani P, Co M, Ramesh T, Akhter N, Iliescu C, Palaskas N, et al. Differentiation of cardiac masses by cardiac magnetic resonance imaging. Curr Cardiovasc Imaging Rep. 2020;13(1)10.1007/s12410-019-9522-4.

[136] Yin H, Mao W, Tan H, Zhu N, Wan Q, Shi J, et al. Role of ^18^F-FDG PET/CT imaging in cardiac and pericardial masses. J Nucl Cardiol. 2022;29(3):1293–303. 10.1007/s12350-020-02510-9. 33462788 10.1007/s12350-020-02510-9

[137] Bernhard B, Gräni C. 18F-FDG PET/CT imaging in the workup of cardiac and pericardial masses. J Nucl Cardiol. 2022;29(6):3466–8. 10.1007/s12350-021-02539-4. 33604789 10.1007/s12350-021-02539-4

[138] Rahbar K, Seifarth H, Schäfers M, Stegger L, Hoffmeier A, Spieker T, et al. Differentiation of malignant and benign cardiac tumors using 18F-FDG PET/CT. J Nucl Med. 2012;53(6):856–63. 10.2967/jnumed.111.095364. 22577239 10.2967/jnumed.111.095364

[139] Nensa F, Tezgah E, Poeppel TD, Jensen CJ, Schelhorn J, Köhler J, et al. Integrated 18F-FDG PET/MR imaging in the assessment of cardiac masses: a pilot study. J Nucl Med. 2015;56(2):255–60. 10.2967/jnumed.114.147744. 25552667 10.2967/jnumed.114.147744

[140] Aghayev A, Cheezum MK, Steigner ML, Mousavi N, Padera R, Barac A, et al. Multimodality imaging to distinguish between benign and malignant cardiac masses. J Nucl Cardiol. 2022;29(4):1504–17. 10.1007/s12350-021-02790-9. 34476778 10.1007/s12350-021-02790-9

[141] Mikail N, Males L, Hyafil F, Benali K, Deschamps L, Brochet E, et al. Diagnosis and staging of cardiac masses: additional value of CMR with ^18^F-FDG-PET compared to CMR with CECT. Eur J Nucl Med Mol Imaging. 2022;49(7):2232–41. 10.1007/s00259-022-05709-9. 35247063 10.1007/s00259-022-05709-9

[142] Andrews JPM, Trivieri MG, Everett R, Spath N, MacNaught G, Moss AJ, et al. 18F-fluoride PET/MR in cardiac amyloid: a comparison study with aortic stenosis and age- and sex-matched controls. J Nucl Cardiol. 2022;29(2):741–9. 10.1007/s12350-020-02356-1. 33000405 10.1007/s12350-020-02356-1 PMC8993737

[143] Martineau P, Finnerty V, Giraldeau G, Authier S, Harel F, Pelletier-Galarneau M. Examining the sensitivity of 18F-NaF PET for the imaging of cardiac amyloidosis. J Nucl Cardiol. 2021;28(1):209–18. 10.1007/s12350-019-01675-2. 30834499 10.1007/s12350-019-01675-2

[144] Abulizi M, Sifaoui I, Wuliya-Gariepy M, Kharoubi M, Israël JM, Emsen B, et al. ^18^F-sodium fluoride PET/MRI myocardial imaging in patients with suspected cardiac amyloidosis. J Nucl Cardiol. 2021;28(4):1586–95. 10.1007/s12350-019-01885-8. 31512197 10.1007/s12350-019-01885-8

[145] Baratto L, Park SY, Hatami N, Gulaka P, Vasanawala S, Yohannan TK, et al. ^18^F-florbetaben whole-body PET/MRI for evaluation of systemic amyloid deposition. EJNMMI Res. 2018;8(1):66. 10.1186/s13550-018-0425-1. 30043115 10.1186/s13550-018-0425-1 PMC6057864

[146] Bi X, Xu B, Liu J, Wang G, An J, Zhang X, et al. Diagnostic value of ^11^C-PIB PET/MR in cardiac amyloidosis. Front Cardiovasc Med. 2022;9:830572. 10.3389/fcvm.2022.830572. 35369284 10.3389/fcvm.2022.830572 PMC8966842

[147] Wisenberg G, Thiessen JD, Pavlovsky W, Butler J, Wilk B, Prato FS. Same day comparison of PET/CT and PET/MR in patients with cardiac sarcoidosis. J Nucl Cardiol. 2020;27(6):2118–29. 10.1007/s12350-018-01578-8. 30603887 10.1007/s12350-018-01578-8 PMC7749056

[148] Sgard B, Brillet PY, Bouvry D, Djelbani S, Nunes H, Meune C, et al. Evaluation of FDG PET combined with cardiac MRI for the diagnosis and therapeutic monitoring of cardiac sarcoidosis. Clin Radiol. 2019;74(1):81.e9-81.e18. 10.1016/j.crad.2018.09.015. 30482560 10.1016/j.crad.2018.09.015

[149] Schneider S, Batrice A, Rischpler C, Eiber M, Ibrahim T, Nekolla SG. Utility of multimodal cardiac imaging with PET/MRI in cardiac sarcoidosis: implications for diagnosis, monitoring and treatment. Eur Heart J. 2014;35(5):312. 10.1093/eurheartj/eht335. 23975480 10.1093/eurheartj/eht335

[150] Wicks EC, Menezes LJ, Barnes A, Mohiddin SA, Sekhri N, Porter JC, et al. Diagnostic accuracy and prognostic value of simultaneous hybrid 18F-fluorodeoxyglucose positron emission tomography/magnetic resonance imaging in cardiac sarcoidosis. Eur Heart J Cardiovasc Imaging. 2018;19(7):757–67. 10.1093/ehjci/jex340. 29319785 10.1093/ehjci/jex340

[151] Greulich S, Gatidis S, Gräni C, Blankstein R, Glatthaar A, Mezger K, et al. Hybrid cardiac magnetic resonance/fluorodeoxyglucose positron emission tomography to differentiate active from chronic cardiac sarcoidosis. JACC Cardiovasc Imaging. 2022;15(3):445–56. 10.1016/j.jcmg.2021.08.018. 34656480 10.1016/j.jcmg.2021.08.018

[152] Tawakol A, Fayad ZA, Mogg R, Alon A, Klimas MT, Dansky H, et al. Intensification of statin therapy results in a rapid reduction in atherosclerotic inflammation: results of a multicenter fluorodeoxyglucose-positron emission tomography/computed tomography feasibility study. J Am Coll Cardiol. 2013;62(10):909–17. 10.1016/j.jacc.2013.04.066. 23727083 10.1016/j.jacc.2013.04.066

[153] Joshi NV, Vesey AT, Williams MC, Shah AS, Calvert PA, Craighead FH, et al. 18F-fluoride positron emission tomography for identification of ruptured and high-risk coronary atherosclerotic plaques: a prospective clinical trial. Lancet. 2014;383(9918):705–13. 10.1016/S0140-6736(13)61754-7. 24224999 10.1016/S0140-6736(13)61754-7

[154] Tarkin JM, Calcagno C, Dweck MR, Evans NR, Chowdhury MM, Gopalan D, et al. ^68^Ga-DOTATATE PET identifies residual myocardial inflammation and bone marrow activation after myocardial infarction. J Am Coll Cardiol. 2019;73(19):2489–91. 10.1016/j.jacc.2019.02.052. 31097170 10.1016/j.jacc.2019.02.052 PMC6525109

[155] Hyafil F, Pelisek J, Laitinen I, Schottelius M, Mohring M, Döring Y, et al. Imaging the cytokine receptor CXCR4 in atherosclerotic plaques with the radiotracer ^68^Ga-Pentixafor for PET. J Nucl Med. 2017;58(3):499–506. 10.2967/jnumed.116.179663. 27789718 10.2967/jnumed.116.179663

[156] Li X, Yu W, Wollenweber T, Lu X, Wei Y, Beitzke D, et al. [^68^Ga]Pentixafor PET/MR imaging of chemokine receptor 4 expression in the human carotid artery. Eur J Nucl Med Mol Imaging. 2019;46(8):1616–25. 10.1007/s00259-019-04322-7. 31004184 10.1007/s00259-019-04322-7 PMC6584241

[157] Hyafil F, Schindler A, Sepp D, Obenhuber T, Bayer-Karpinska A, Boeckh-Behrens T, et al. High-risk plaque features can be detected in non-stenotic carotid plaques of patients with ischaemic stroke classified as cryptogenic using combined (18)F-FDG PET/MR imaging. Eur J Nucl Med Mol Imaging. 2016;43(2):270–9. 10.1007/s00259-015-3201-8. 26433367 10.1007/s00259-015-3201-8

[158] Blockmans D, Coudyzer W, Vanderschueren S, Stroobants S, Loeckx D, Heye S, et al. Relationship between fluorodeoxyglucose uptake in the large vessels and late aortic diameter in giant cell arteritis. Rheumatology (Oxford). 2008;47(8):1179–84. 10.1093/rheumatology/ken119. 18515868 10.1093/rheumatology/ken119

[159] Cerne JW, Liu S, Umair M, Pathrose A, Moore JE, Allen BD, et al. Combined modality PET/MR for the detection of severe large vessel vasculitis. Eur J Hybrid Imaging. 2022;6(1):16. 10.1186/s41824-022-00136-3. 35965266 10.1186/s41824-022-00136-3 PMC9376186

[160] Padoan R, Crimì F, Felicetti M, Padovano F, Lacognata C, Stramare R, et al. Fully integrated [18F]FDG PET/MR in large vessel vasculitis. Q J Nucl Med Mol Imaging. 2022;66(3):272–9. 10.23736/S1824-4785.19.03184-4. 31602964 10.23736/S1824-4785.19.03184-4

[161] Rischpler C, Nekolla SG, Dregely I, Schwaiger M. Hybrid PET/MR imaging of the heart: potential, initial experiences, and future prospects. J Nucl Med. 2013;54(3):402–15. 10.2967/jnumed.112.105353. 23404088 10.2967/jnumed.112.105353

